# Long‐Term Outcomes on Pallidal Neurostimulation for Dystonia: A Controlled, Prospective 10‐Year Follow‐Up

**DOI:** 10.1002/mds.30130

**Published:** 2025-02-05

**Authors:** Patricia Krause, Philipp Mahlknecht, Inger Marie Skogseid, Frank Steigerwald, Günther Deuschl, Richard Erasmi, Alfons Schnitzler, Tobias Warnecke, Jörg Müller, Werner Poewe, Gerd‐Helge Schneider, Jan Vesper, Nils Warneke, Wilhelm Eisner, Thomas Prokop, Jan‐Uwe Müller, Jens Volkmann, Andrea A. Kühn, for the Deep‐Brain Stimulation for Dystonia Study Group, Maximilian Mehdorn, Patricia Krause, Doreen Gruber, Anatol Kivi, Andreas Kupsch, Bianca Müller, Gerd‐Helge Schneider, Alfons Schnitzler, Volker Sturm, Lars Timmermann, Jürgen Voges, Lars Wojtecki, Guido Nikkah, Markus Pinsker, Thomas Prokop, Jan Vesper, Manja Kloss, Martin Krause, Volker Tronnier, Wilhelm Eisner, Thomas Fiegele, Jörg Müller, Sasha Hering, Werner Poewe, Günther Deuschl, Jan Herzog, Marcus O Pinsker, Monika Pötter‐Nerger, Frank Steigerwald, Hans‐Werner Boothe, Angela Brentrup, Juliane Vollmer‐Haase, Inger Marie Skogseid, Geir Ketil Roeste, Jan‐Uwe Müller, Matthias Wittstock, Alexander Wolters, Joseph Classen, Markus Naumann, Alex Schramm, Jens Volkmann, Andrea A. Kühn

**Affiliations:** ^1^ Movement Disorder and Neuromodulation Unit, Charité, University Medicine Berlin Berlin Germany; ^2^ Department of Neurology Medical University Innsbruck Innsbruck Austria; ^3^ Department of Neurology Oslo University Hospital Oslo Norway; ^4^ Department of Neurology University of Würzburg Würzburg Germany; ^5^ Department of Neurology Christian‐Albrechts‐University Kiel Germany; ^6^ Institute of Clinical Neuroscience and Medical Psychology, and Department of Neurology, Medical Faculty Heinrich‐Heine‐University Düsseldorf Germany; ^7^ Department of Neurology University of Münster Münster Germany; ^8^ Department of Neurology and Rehabilitation Klinikum Osnabrück, Academic Teaching Hospital of the University of Münster Osnabrück Germany; ^9^ Department of Neurology Vivantes Klinikum Spandau Berlin Germany; ^10^ Department of Neurosurgery Charité, University Medicine Berlin Berlin Germany; ^11^ Department of Stereotactic and Functional Neurosurgery University of Düsseldorf Düsseldorf Germany; ^12^ Department of Neurosurgery University of Münster Münster Germany; ^13^ Department of Neurosurgery Medical University Innsbruck Innsbruck Austria; ^14^ Division of Stereotactic and Functional Neurosurgery University of Freiburg Freiburg Germany; ^15^ Department of Neurology Universitätsmedizin Greifswald Greifswald Germany

**Keywords:** dystonia, deep brain stimulation, neurostimulation, long‐term follow‐up, DBS response

## Abstract

**Background:**

Pallidal neurostimulation is an effective treatment for severe isolated dystonia, but long‐term data from clinical trials are lacking.

**Objectives:**

To evaluate long‐term efficacy and safety of pallidal neurostimulation in patients with isolated generalized or segmental dystonia.

**Methods:**

Extension study of the prospective multicenter trial (n = 40; July 2002 to May 2004), all patients received effective stimulation and underwent regular follow‐up. The 10‐year follow‐up (n = 31) included Burke–Fahn–Marsden Dystonia Rating Scale (BFMDRS) motor and disability score, Beck Depression Inventory, Beck Anxiety Inventory, and Mattis Dementia Rating Scale. Primary and secondary endpoints compared motor symptoms, disability scores, mood, and cognition changes.

**Results:**

Thirty‐one patients (12 female), aged 23–72 years, completed the 10‐year study extension. Per protocol analysis showed sustained significant improvement in BFMDRS motor scores at 10 years compared with baseline, without significant change from the 6‐month or 5‐year follow‐up. On average, motor scores decreased by 25.3 ± 5.2 points at 10 years (*P* < 0.0001; 56% improvement). Individual outcomes varied, with 27 responders (≥25% improvement; mean improvement 65.2 ± 21.4%) and 13 non‐responders compared with baseline. Sustained improvements were seen in disability, mood, and anxiety scores. Cognition remained stable.

**Conclusions:**

This study presents the longest prospective, multicenter follow‐up of pallidal neurostimulation in generalized and segmental dystonia. Two‐thirds of patients showed strong and stable long‐term improvements of dystonia, confirming sustained efficacy and safety over 10 years in treatment‐refractory dystonic patients. However, one‐third experienced primary (3/40) or secondary (10/40) treatment failure. Diagnostic advances, including genetic testing, and technological progress in pallidal neurostimulation may help to reduce the non‐responder rates in the future. © 2025 The Author(s). *Movement Disorders* published by Wiley Periodicals LLC on behalf of International Parkinson and Movement Disorder Society.

Isolated dystonia is a movement disorder characterized by involuntary muscle contractions causing twisting and sometimes tremulous movements.^1^ Isolated generalized dystonia (GD) often begins during childhood, while isolated segmental dystonia (SD), involving contiguous body regions, usually appears in adulthood and does not tend to spread.^1^ Dystonia significantly impacts everyday life and social participation,^2^ and may result in chronic motor impairment, pain, and social withdrawal in young patients with a regular life expectancy and lifelong need for therapy. Oral medications often provide insufficient symptom relief, and botulinum toxin injections are limited to less complex clinical presentations.^3^, ^4^ Deep brain stimulation (DBS) of the globus pallidus internus (GPi) has evolved into a widely available treatment in medically refractory dystonia.^3^, ^5^, ^6^ Long‐term follow‐up (FU) in clinical trials has been reported for 3 years^7^ and 5 years^8^ showing sustained motor improvement, reduced disability, and enhanced quality of life (QoL), along with reduced depression and anxiety scores. Numerous retrospective and uncontrolled studies support the long‐term benefits of pallidal stimulation in motor function, disability, pain reduction, and QoL.^7^, ^9^, ^10^, ^11^, ^12^, ^13^, ^14^ Predictive factors for pallidal DBS efficacy in isolated dystonia include shorter disease duration,^15^, ^16^, ^17^ younger age at onset and surgery,^11^ genetic subtype,^18^, ^19^, ^20^ and distal body distribution of dystonic symptoms.^8^, ^21^ Conversely, the presence of fixed musculoskeletal deformities^11^, ^16^, ^17^ and reduced GPi volume^22^ may negatively impact DBS outcome. Optimal lead placement is crucial for optimal motor response, although the definition of the pallidal ‘hot spot’ in dystonia can be challenging.^23^, ^24^ Approximately 20% of isolated dystonia patients are primary non‐responders (defined as ≤25% or 30% motor improvement).^5^, ^6^, ^25^ Concerns also exist about secondary worsening of initial responders.^10^ Questions regarding the risk‐to‐benefit balance, therapy sustainability, or secondary worsening after long‐term stimulation are of significant clinical interest in a young patient group with ongoing professional and personal demands during lifelong therapy.^7^, ^10^, ^13^

Here, we present the most extended FU on the effects of GPi‐DBS, including prospective assessments of motor and disability scores, non‐motor symptoms, and adverse events. This follows our prospective, blinded, and sham‐controlled trial for primary GD or SD,^5^ which was amended by a 10‐year FU visit after completion of the initial 5‐year open‐label extension phase.

## 1 Methods

### 1.1 Study Design and Patients

In the parent trial,^5^ 40 patients (age 14–75 years) with severe medically refractory idiopathic GD or SD were randomized to either sham‐ or neurostimulation in the GPi bilaterally for 3 months. This was followed by an open‐label active stimulation period evaluated at 6 months after treatment onset. Ten neurological centers in Austria, Germany, and Norway recruited patients between 2002 and 2004. Details on the original study protocol including randomization, enrollment, and blinding can be found in the parent trial,^5^ in clinicalTrials.gov (NCT00142259) and the flow diagram (Supplementary Material 1). Exclusion criteria encompassed previous brain surgery, cognitive impairment (Mattis Dementia Rating Scale [MDRS] <120 points), moderate‐to‐severe depression (Beck Depression Inventory [BDI] >25 points), significant brain atrophy, and increased surgical risk due to medical or psychiatric comorbidities. Thirty‐eight patients agreed to annual FU visits for up to 5 years after neurostimulation activation.^8^ The FU period was later extended to 10 years by protocol amendment with approval from the Institutional Review Board of Kiel University's medical faculty in 2012. The 10‐year visit protocol was identical to the 5‐year visit. The Kiel and Berlin sites collaborated on data monitoring and management. Ethical committees of each participating center approved the study protocol extension (Supplementary Material 2), and all patients provided informed consent for inclusion in the extension period (D450/12) of the original study (A121/02). The original trial is registered in ClinicalTrials.gov (NCT00142259).

### 1.2 Outcome Measures

Baseline and FU assessments included evaluation of dystonia severity and disability using the Burke–Fahn–Marsden Dystonia Rating Scale (BFMDRS).^26^ Non‐motor symptoms like mood disturbances and anxiety were evaluated using the Beck Depression and Anxiety Inventory (BDI and BAI).^27^ Cognitive testing was done by means of the MDRS.^28^ Severity of dystonia and pain were rated with the use of a visual analog scale (VAS) with scores ranging from 0 to 10, with higher scores indicating greater severity. These open‐label assessments, conducted by experienced neurologists, were videotaped and done under continuous stimulation. Drug intake reflected individual FU recommendations. Device‐related side effects and (serious) adverse events were monitored, with new or worsened pre‐existing symptoms classified as adverse event (AE) compared with 5‐year FU. Serious adverse events (SAEs) included critical medical conditions or outcomes requiring intervention. Regular device replacement due to battery depletion was not classified as a (severe) AE. To explore possible causes of DBS non‐response, we re‐evaluated updated genetic information, additional clinical features, patient history, lead placement, stimulation settings, and system integrity, where available.

### 1.3 Statistical Analysis

The primary study endpoint was dystonia severity measured by the BFMDRS motor score. We assessed long‐term treatment effects at the 5‐ and 10‐year FU visits compared with baseline and the 6‐month visit post‐stimulation. Analysis followed an intention‐to‐treat (ITT) approach, including all initially randomized patients with missing values imputed using the restricted maximum likelihood estimation (REML) method in a multivariate model and per protocol analysis. Secondary analysis defined a clinically meaningful response as ≥25% improvement in the BFMDRS motor score. Secondary endpoints included the BFMDRS disability score. Other outcomes were exploratory and analyzed per protocol without imputation of missing values. Long‐term safety was evaluated by examining the number and severity of reported AEs. Wilcoxon rank‐sum test for matched pairs was used to compare 5‐ and 10‐year scores with baseline and 6‐month scores. The *χ* ^2^ test was used for categorical data. Significance was set at *P* < 0.05 (two‐tailed) after adjusting for multiple comparisons. JMP statistical package (version 8.0.2) was used for all statistical analyses. Results are presented as mean ± standard deviation (STD).

## 2 Data Sharing

We welcome inquiries from fellow researchers and interested parties to facilitate further exploration and knowledge dissemination in this field. The original data used in this publication including individual stimulation settings of each patient are available for further insight upon request. To access the dataset please contact the corresponding author.

## 3 Results

Thirty‐one of the initial 40 patients agreed to participate in and completed the 10‐year study extension after 32 patients had attended the previous 5‐year FU.^8^

### 3.1 Motor Symptoms and Disability

**FIG. 1:**
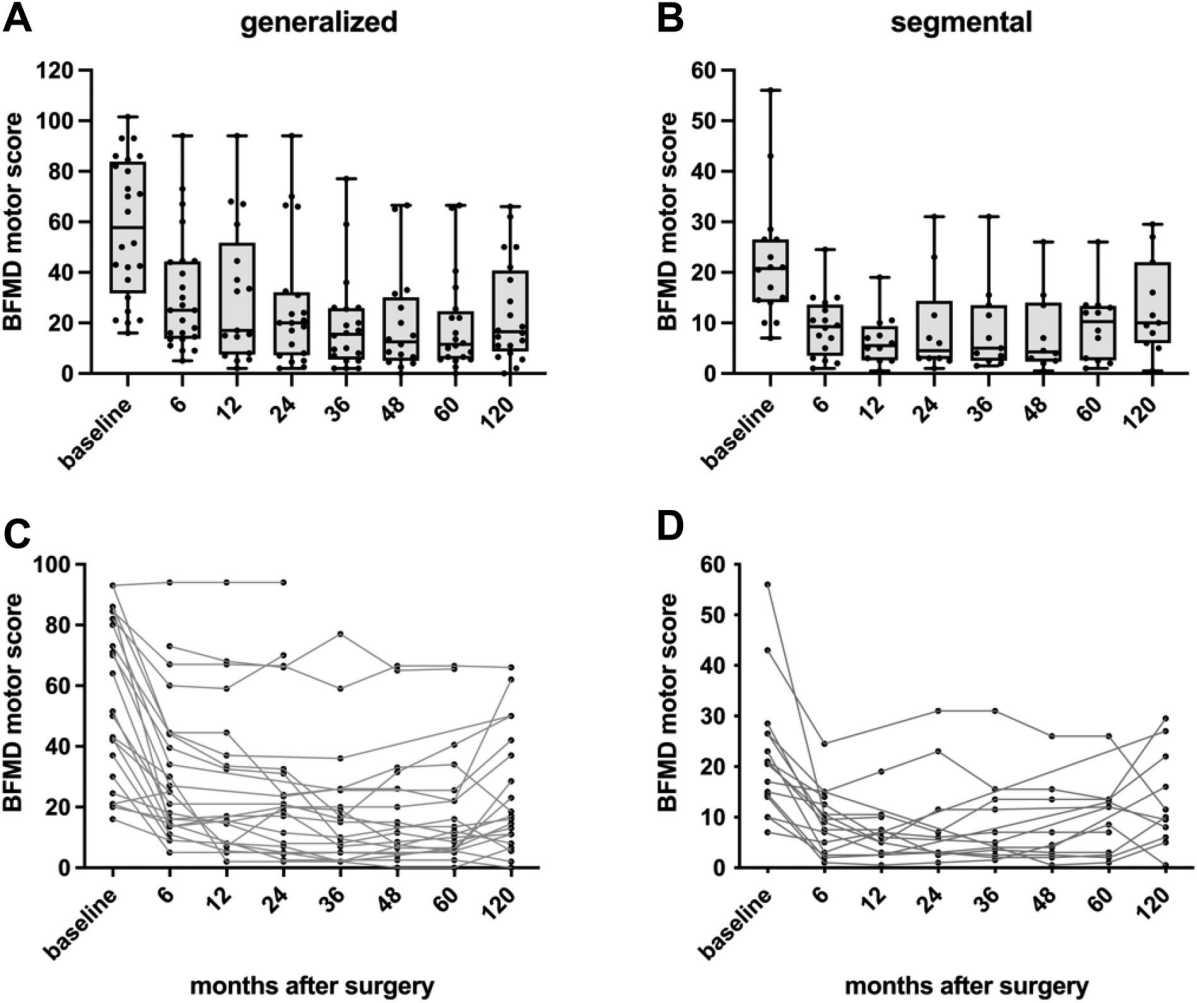
(**A**, **B**) The whisker plots visually represent the distribution of absolute Burke–Fahn–Marsden Disease (BFMD) motor scores at baseline and after 6, 12, 24, 36, 48, 60, and 120 months of continuous pallidal neurostimulation separated for generalized (GD) and segmental dystonia (SD) patients along with individual datapoints for each patient. The whiskers extend from the minimum to the maximum values, showing the full range of the data. Each point represents an individual patient's data, providing a detailed view of the distribution. The box marks the interquartile range, located between the 25th and 75th percentiles, and encapsulates the middle 50% of the data. The line within the box denotes the median, indicating the central value of the dataset. (**C**, **D**) The scatter plots provide visual representation of individual BFMD motor score trajectories from all patients with GD and SD dystonia at baseline and after 6, 12, 24, 36, 48, 60, and 120 months of continuous pallidal neurostimulation.

The ITT analysis (including all 40 patients) showed significant group mean reductions in BFMDRS motor scores at 6 months, 5 and 10 years (*P* < 0.0001) compared with baseline. In the per protocol cohort (n = 31), a significant dystonia score reduction (*P* < 0.0001) was sustained at FU, with a mean decrease of 25.3 points (standard error [SE] ± 5.2) corresponding to a 56% improvement after 10 years. There was no difference in the relative motor improvement between patients with generalized (n = 24) and segmental (n = 16) dystonia after 6 months, and 5 or 10 years, although absolute score values were higher in GD versus SD because of more body regions affected (see Fig. 1). Total BFMDRS disability score in the group analyzed per protocol was significantly decreased at each FU, with a mean reduction of 3.5 ± 1.5 points (n = 31, *P* = 0.02) 10 years after baseline. Patients reported a significant reduction in motor symptoms through the VAS scores, from of 7.0 ± 1.7 points at baseline to 4 ± 2.1 points at the 10‐year FU (*P* < 0.0001). Clinicians' scores declined from 6.6 ± 2.0 points at baseline to 3.5 ± 1.2 points at last evaluation (*P* < 0.0001) (Table 1).

**TABLE 1:** Effect of pallidal neurostimulation on dystonia‐associated symptoms and disability (the Burke–Fahn–Marsden Dystonia Rating Scale [BFMDRS] represents the symptom severity of dystonia)

	Before surgery	6 Months	3 Years	5 Years	10 years	p Value	p Value	p Value	p Value
	n Mean (STD)	n Mean (STD)	n Mean (STD)	n Mean (STD)	n Mean (STD)	(10 years vs. baseline)	(10 years vs. 6 months)	(10 years vs. 3 years)	(10 years vs. vs. 5 years)
BFMDRS motor score (ITT)	40–43.3 (28.6)	40–22.6 (21.2)	40–18.0 (21.6)	40–17.9 (21.2)	40–21.6 (22.5)	<0.0001	0.8407	0.4812	0.4659
BFMDRS motor score (PP)									
Total	40–43.3 (28.6)	40–22.6 (21.2)	31–15.6 (17.0)	32–14.3 (15.8)	31–18.1 (17.3)	<0.0001	0.3964	0.6533	0.495
Face (eyes and mouth)	40–2.8 (3.1)	40–1.7 (3.0)	31–0.9 (1.5)	31–1.0 (1.6)	31–0.8 (1.2)	0.0012	0.1664	0.9379	0.8558
Speech and swallowing	40–3.8 (4.9)	40–3.1 (4.9)	31–2.2 (3.8)	31–2.5 (3.2)	31–2.5 (2.8)	0.2196	0.5763	0.7711	0.936
Axial (neck and trunk)	40–15.2 (5.5)	39–6.7 (4.7)	31–4.3 (4.1)	31–4.6 (4.0)	31–5.2 (4.3)	<0.0001	0.1684	0.7228	0.4334
Extremities	39–22.2 (20.7)	39–11.5 (15.5)	31–8.3 (12.5)	31–6.6 (11.4)	31–9.7 (13.7)	0.0008	0.6142	0.7228	0.4334
BFMDRS disability score									
Total	40–10 (6.6)	40–6.1 (6.0)	31–5.9 (6.1)	32–5.3 (5.8)	31–6.5 (4.5)	0.0196	0.7779	0.7109	0.4154
Speech	40–1.5 (1.3)	40–1.2 (1.3)	31–1.1 (1.3)	32–1.2 (1.3)	31–1.4 (1.0)	0.5781	0.6128	0.427	0.6727
Writing	40–1.7 (1.1)	40–1.3 (1.2)	31–1.1 (1.1)	32–10 (1.2)	31–0.9 (0.8)	0.0032	0.1628	0.468	0.8043
Feeding	40–1.4 (1.2)	40–0.7 (1.0)	31–0.5 (0.9)	32–0.5 (0.9)	31–0.7 (0.8)	0.0038	0.9922	0.391	0.4968
Eating and swallowing	40–0.8 (1.0)	40–0.4 (0.6)	31–0.3 (0.7)	32–0.3 (0.5)	31–0.5 (0.8)	0.1891	0.5139	0.3056	0.2774
Hygiene	40–1.2 (1.2)	40–0.6 (1.1)	31–0.5 (1.0)	32–0.5 (1.0)	31–0.6 (0.8)	0.0091	0.8414	0.8137	0.7694
Dressing	40–1.2 (1.2)	40–0.6 (1.1)	31–0.6 (1.1)	32–0.5 (1.0)	31–0.7 (0.8)	0.0974	0.6114	0.5665	0.4527
Walking	40–2.3 (1.5)	40–1.4 (1.5)	31–1.9 (2.2)	32–1.3 (1.4)	31–1.8 (1.6)	0.2504	0.3186	0.7653	0.2499
Visual analog scale									
Dystonia severity (patient)	40–7.0 (1.7)	38–3.7 (2.2)	29–3.2 (2.2)	31–3.4 (2.2)	30–4.0 (2.1)	<0.0001	0.5308	0.193	0.2601
Pain severity (patient)	40–4.6 (2.7)	38–1.6 (1.8)	29–1.7 (1.8)	31–2.2 (2.5)	28–3.1 (2.4)	0.0065	0.0116	0.0195	0.1550
Dystonia severity (physician)	40–6.6 (2.0)	39–3.5 (1.9)	29–3.4 (2.0)	32–3.4 (2.0)	29–3.5 (1.8)	<0.0001	0.9673	0.7558	0.8375

The total BFMDRS motor score is the sum of individual scores for each body region ranging from 0 to 120 points (higher values indicating maximal symptoms). The individual score is a product of the *severity factor* ranging from 0 to 4 with, for example, 0 indicating no dystonia and 4 maximal dystonia and the *provoking factor ranging* from 0 to 4 with, for example, 1 indicating onset of dystonia during special tasks and 4 indicating dystonia at rest. The total disability score, ranging from 0 to 30 points, is the sum of individual ratings for seven activities: speech (0 indicating easy understanding and 4 indicating almost complete anarthria); handwriting (0 indicating legible handwriting and 4 indicating inability to grasp); dependence as to hygiene, dressing, and feeding (0 indicating independence and 4 indicating complete dependence); swallowing (0 indicating normal ability and 4 indicating marked difficulty of swallowing soft food and liquids); and walking (0 indicting normal gait and 6 indicating dependence on wheelchair). Scores on the visual analog scale ranging from 0 (no complaints) to 10 (maximal complaints) represent the subjective impression of dystonia and/or pain severity from the patients' or physicians' perspective.

Abbreviations: STD, standard deviation; BFMDRS, Burke–Fahn–Marsden Dystonia Rating Scale; ITT, intention to treat; PP, per protocol.

#### 3.1.1 Responder/Non‐Responder Analysis

In the ITT population, 27 patients were classified as responders (≥25% improvement) with a mean improvement of 68.1 ± 20.3% (range 31.5–98.0%) compared with baseline. Thirteen patients were classified as non‐responders with a mean motor change of 8.5 ± 27.3% (ranging from 60% deterioration to a 22% improvement in BFMDRS) at last available FU (Table 2). Among these, three were primary non‐responders (PNR) at 6 months, and another 10 patients became secondary non‐responders (SNR) before the subsequent follow‐ups at 1 year (n = 1), 2 (n = 1), 5 years (n = 3), and 10 years (n = 5).

**TABLE 2:** Response of motor impairment throughout the study

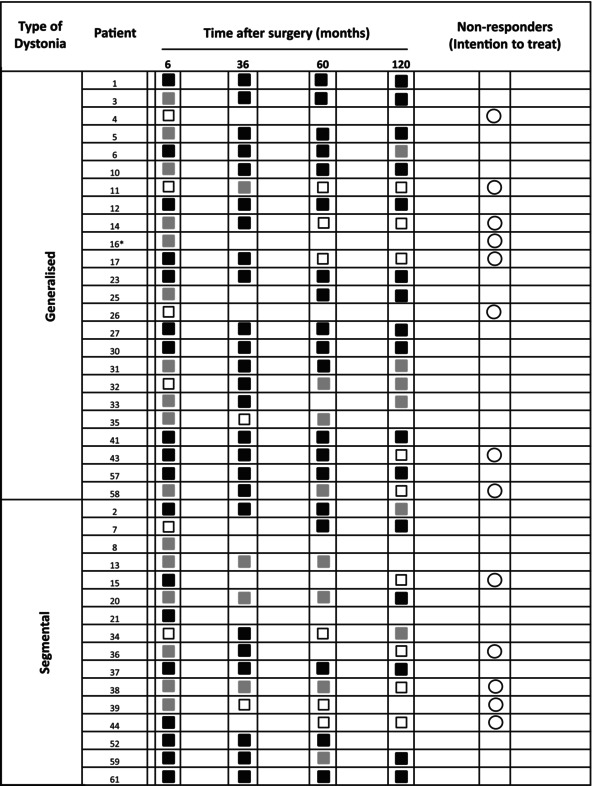

Color‐coded squares symbolize response to pallidal neurostimulation over time with, for example, a black square indicating ≥50%, a grey square indicating 25–50%, and a white square indicating <25% motor improvement in the Burke–Fahn–Marsden Dystonia Rating Scale motor symptoms in comparison to baseline scores. Non‐responders (white circles) in the intention‐to‐treat (ITT) cohort were defined to show <25% improvement or worsening of dystonia motor score at last follow‐up.

* Patient 16 lost therapeutic response at the 24‐month follow‐up.

A retrospective chart and video review by two dystonia experts (A.A.K. and J.V.) revealed one or several potential causes of therapeutic non‐response in 12/13 non‐responders: three patients had clinical signs not compatible with isolated dystonia at baseline and must be considered selection failures. THAP1‐gene mutation, known for a more variable DBS response, was found in one patient with severe dystonic posturing at baseline and a 26‐year disease duration before surgery (more details in Krause et al., 2015).^12^ Three primary and three secondary non‐responders had suspected or confirmed lead misplacement documented by postoperative imaging (one improved after revision surgery). Moreover, in five non‐responders optimal programming was limited by adverse effects such as stimulation‐induced bradykinesia or capsular effects affecting dystonia ratings. Further details on individual clinical, diagnostic, and stimulation findings that could have contributed to therapeutic DBS failure in the non‐responder group can be found in Table 3.

**TABLE 3:** Summary of clinical and paraclinical findings for possible reasons of deep brain stimulation failure in the non‐responder cohort

Patient #	SD/GD	PNR	SNR	Items of retrospective chart and video review				Expert judgement of causes for non‐response (certainty)
				Clinical features and past medical history	Genetic diagnosis	Clinical confounders of postoperative dystonia rating	Suspected or documented DBS failure (surgical or programming)	
4	GD	X		Severe bulbar involvement, broad based unsteady gait	TOR1a‐negative	–	Bilaterally misplaced electrodes (2011 system explantation without re‐implantation)	Non‐response due to lead misplacement and possible genetic status
11	GD	X		Onset in early childhood (3 years); wheelchair‐bound, truncal hypotonia, pathological laughter	NA	‐	misplaced right electrode on postoperative imaging	Non‐response due to patient selection failure (non‐isolated dystonia) and unilateral lead misplacement (definite)
14	GD		X	–	NA	Generalized bradykinesia and increased muscle tone	Bilateral high stimulation amplitudes	Non‐response due to unilateral lead misplacement and unreliable FU dystonia rating due to overstimulation (possible)
16	GD		X	–	THAP1‐DYT‐mutation	–	–	Non‐response due to genetic status (possible)
17	GD		X	Intention tremor, myoclonus, ataxia	Dystonia gene panel and exome negative; genome running	Unilaterally increased muscle tone, resolved after DBS adjustment (2013)	Unilateral high stimulation amplitude	Non‐response due to patient selection failure (non‐isolated dystonia) and suspected unilateral lead misplacement (definite)
26	GD	X		_	TOR1A‐DYT‐mutation	–	Bilaterally misplaced electrodes, significant improvement after electrode repositioning (BFMDRS‐M before/after: 23/6 points) after study exit at 6 months FU	Non‐response due to lead misplacement (definite)
43	GD		X	Significant laryngeal and cervical dystonia with skipping and stiff‐legged gait	NA		wound revision and system ex‐, re‐implantation before 60 months FU	Non‐response possibly related to complex dystonic phenotype (no genetic testing available) and unreliable FU dystonia rating due to overstimulation (possible)
58	GD		X	Second born twin with reported hypoxia and reanimation at birth; intention tremor, ataxia, onset in early childhood with delay of motor milestones	Negative for TOR1A‐DYT‐mutation		Misplaced right electrode	Non‐response due to patient selection failure (non‐isolated dystonia, possible cerebral palsy) and lead misplacement (definite)
15	SD		X	–	NA	Stimulation induced dysphonia; improvement of dystonic movements not captured by BFMDRS spasticity due to symptomatic cervical myelopathy at 10 years FU	‐	Non‐response due to unreliable FU dystonia rating due to overstimulation and underrating of mobile dystonia improvement (definite)
36	SD		X	–	NA	stimulation induced dysarthria and dysphagia at last FU, bradykinesia	Bilateral high stimulation amplitudes; no postoperative imaging available	Non‐response due to unreliable FU dystonia rating due to overstimulation (possible)
38	SD		X	THAP1‐DYT‐mutation‐PT (severe craniocervical and truncal involvement)	Negative for TOR1A‐DYT‐mutation, no other tests	Clinically generalized instead of segmental	System infection with ex‐ and re‐implantation between 5‐ and 10‐months FU	Non‐response due to surgical DBS failure and/or to genetic status (possible)
39	SD		X	–	NA	‐–	–	Non‐response unexplained
44	SD		X		NA	Lower limb bradykinesia and gait impairment at 10‐year FU	–	Non‐response due to unreliable FU dystonia rating due to overstimulation (possible)

Abbreviations: SD, segmental dystonia; GD, generalized dystonia; PNR, primary non‐responder; SNR, secondary non‐responder; PT, phenotype; DYT, dystonia; DBS, deep brain stimulation; FU, follow‐up; BFMDRS, Burke–Fahn–Marsden Disease Rating Scale; NA, not applicable.

### 3.2 Non‐Motor Symptoms and Medication

Mean baseline anxiety scores (12.6 ± 10.7 points; n = 38) improved to 9.9 ± 10.5 points (*P* = 0.2; n = 31) after 10 years. Depressive symptoms with a mean baseline score of 9.0 ± 6.9 points (n = 38) remained reduced to 6.6 ± 8 points (*P* = 0.2; n = 31) at 10‐year FU. MDRS at last FU (135 ± 25; range 103–144) did not differ significantly from baseline values (136.1 ± 15.3 points; range 121–144, *P* = 0.8). Patient pain ratings via VAS improved significantly, decreasing from 4.6 ± 2.7 points at baseline to 3.1 ± 2.4 points at 10‐year FU (*P* = 0.007), as shown in Supplementary Material 3. After 10 years, 12 of 31 patients (39%) required additional medication for dystonic symptoms and pain, compared with 24 of 40 patients (60%) at baseline. Nine patients still used low‐dose benzodiazepines daily or as needed and five received regular botulinum toxin injections for remaining focal dystonia (primarily neck or extremities) (for details see Supplementary Material 3).

### 3.3 Stimulation Parameters

Twenty‐three patients received single monopolar stimulation, and the remaining eight patients received double monopolar stimulation. Mean frequency (163.9 ± 34.4 Hz; range 80–230 Hz), pulse width (119.1 ± 45.9 μs; range 60–270 μs), and amplitude (3.3 ± 0.8 V; range 1.7–4.4 V) were similar at 5‐ and 10‐year FU (for details see Supplementary Material 3). Stimulation parameters did not differ significantly between GD and SD patients.

### 3.4 Safety

Between 5‐year FU and 10‐year FU, 13 AEs and 17 SAEs occurred and required surgical intervention in 15 patients. SAEs included two whole‐system infections with consecutive explantation and re‐implantation, two implantable pulse generator (IPG)‐infections, four stimulation malfunctions after IPG changes causing three IPG corrections, and one electrode explantation and re‐implantation due to accidental damage. Main AEs were dysarthria/dysphagia (n = 4), dystonia worsening (n = 5), and paresthesia around the IPG‐pocket (n = 2). Three patients (patients 36, 43, 44) presented stimulation‐related generalized bradykinesia during retrospective analysis of available (n = 27/38) video‐data at the 10‐year follow‐up. One patient (patient 15) with preoperative anterocollis developed cervical myelopathy. SAEs affected GD and SD patients equally. Adverse events were 43% less frequent at 10‐year FU in comparison to the 6‐month or 5‐year observation period. Compared with the first 5 years of neuromodulation, the number of AEs and SAEs during years 5–10 decreased from 28 to 13 and from 21 to 11, respectively. None of the SAEs led to treatment discontinuation (for details see Table 4).

**TABLE 4:** Adverse events and serious adverse events* between time points baseline/6 months, 6 months/5 years, and 5 years/10 years

Serious adverse events*	Baseline to 6 months	6 months to 5 years	5 years to 10 years	Total
Subcutaneous infection	4	3	5	12
Lead dislodgement	1	3	1	5
Lead or extension malfunction	0	5	2	7
Stimulator malfunction	0	3	1	4
inpatient troubleshooting programming	0	2	0	2
Lead repositioning	0	2	1	3
Cervical myelopathy	0	1	1	2
Peripheral denervation surgery	0	1	0	1
Depressive episode and attempted suicide	0	1	0	1
Adverse events				
Dysarthria/dysphagia	5	11	4	20
Worsening of dystonia	2	5	5	12
Transient malfunction /deactivation of IPG	0	6	0	6
Partial lead breakage	1	0	0	1
Dysesthesias	2	2	2	6
Gait disorder	1	1	1	3
Postoperative confusion	1	0	0	1
Depressive episode	0	1	1	2
Frequent yawning	0	1	0	1
Seizure	1	0	0	1
Seroma	1	0	0	1
Stuttering	1	0	0	1
Sleep disorder	1	0	0	1
Facial weakness	1	0	0	1
Omarthrosis	0	1	0	1
Total events	**22**	**49**	**24**	**88**
Total affected patients	**19**	**32**	**17**	

* Requiring hospital admission or lengthening of hospital stay.

## 4 Discussion

We present the first prospective, multicenter, long‐term FU of patients with GD and SD undergoing bilateral GPi‐DBS for up to 10 years. Notably, there was a 56% improvement in dystonia severity after a decade of GPi‐DBS, which was similar to the 65% reduction observed after 5 years in the per protocol population. Nevertheless, 13/40 (33%) patients were classified as primary or secondary non‐responders, due to various reasons discussed below. Overall, GPi‐DBS proved to be a safe long‐term treatment, with fewer (severe) AEs occurring during the 5–10‐year FU period compared with the initial 6 months to 5 years of chronic DBS.

### 4.1 Motor Symptoms, Pain and Disability over 10 Years of Pallidal Neurostimulation

In both patient groups with GD and SD, mean motor score improvement increased up to the 3‐year FU and then stabilized. Despite a mild increase in mean motor scores from 5‐ to 10‐year FU, motor impairment remained significantly reduced compared with baseline in each group. Both the clinical raters and the patients reported significant motor improvements during the 10‐year treatment period, accompanied by a significant reduction in pain levels. Furthermore, disability scores remained stable in the GD cohort, while all individual disability items deteriorated in the SD group. Heterogeneity and clinical characteristics of the baseline study population have been reported in detail previously.^8^ Positive predictors for beneficial neurostimulation include shorter disease duration, younger age at onset, and surgery^11^, ^15^, ^16^, ^17^ as well as better responses in more distal dystonic body regions.^8^, ^21^, ^29^ In our cohort, patients with GD were significantly younger at disease onset and surgery, and presented with higher motor scores mainly involving the extremities and face at baseline. In contrast, SD patients with comparably smaller DBS effects primarily experienced cervical dystonia as a more axial symptom. Additionally, patients with SD exhibited worsening of ‘speech’ in the dystonia motor score. We can only speculate that reduced benefits for cervical symptoms and stimulation‐related side effects such as dysarthria or bradykinesia might have influenced disability scores in SD.

### 4.2 Non‐Responder Analysis

Up to 20% non‐responders of pallidal DBS have been described in several cohorts of isolated dystonia patients with the response threshold set at 25% or 30% improvement in BFMDRS. In our prospective trial, we identified three primary and 10 secondary non‐responders comprising up to 32.5% of the initial cohort. Potential reasons for non‐response in previous studies included poor lead location, fixed deformities, as well as non‐isolated and functional dystonia.^5^, ^25^, ^30^, ^31^ Similarly, in our cohort we identified several reasons for non‐response with non‐isolated dystonia (n = 3) and lead misplacement (n = 7) as main causes. Image‐guided lead positioning was not investigated systematically within the trial and is thus not available for all patients. In accordance with the practices at the time of implantation, all patients underwent intraoperative electrophysiological testing as well as a comprehensive postoperative standardized initial programming algorithm aiming at optimal lead localization and to exclude stimulation‐associated capsular effects. However, four patients were included in an additional non‐responder analysis across 13 experienced DBS centers revealing that electrodes in three of them were suboptimally placed.^30^ In another three patients suboptimal lead localization was found via Lead‐DBS^32^ during DBS optimization at Charité after so far insufficient treatment effects. Beyond the performance of intensive stimulation adjustments, these patients did not wish to undergo electrode revision surgery. Notably, one primary non‐responder (patient 26) did experience significant DBS benefit after lead correction in the extension period.^30^

Conversely, as shown in Table 2, patients 14 and 58 were already only moderately responsive (35–50% = gray square) at the 6‐month FU and, in detail, were actually close to non‐response. They exceeded the 50% threshold once at the 3‐year FU but were rated as <25% responsive at the 5‐year FU and subsequently. Suboptimal lead placement would most likely result in partial and more variable response from the beginning. In contrast, for example, patient 17 initially showed a good response of 58%, and dystonia scores steadily increased only later, starting from the 5‐year FU. According to Table 3, there were growing indications of a non‐isolated dystonia as a possible cause for the worsening of symptoms. The new dystonia classification published in Albanese et al. 10 years after initial patient recruitment for the present study provided a more comprehensive framework for categorizing different forms of dystonia and to enhance precision in diagnosis.^1^ Bearing this in mind, additional clinical features as found in patients 11, 17, and 58 (compare Table 3) suggest the possibility of acquired or inherited neurodegenerative combined dystonia rather than isolated dystonia. Stimulation effects in acquired or combined inherited neurodegenerative dystonia have been reported as less pronounced and highly variable.^33^, ^34^, ^35^ The non‐isolated phenotypes in the aforementioned patients might have had an impact on DBS response. Furthermore, prior studies reported a potential favorable or negative impact of certain gene mutations on DBS benefits.^12^, ^15^, ^18^, ^19^, ^20^, ^36^ Genotype documentation for revising underlying gene mutations was not part of the initial study protocol and remains unclear in a large part of the cohort. However, after re‐evaluation of patients' records and videos, we suggest that factors such as non‐isolated dystonia and less responsive DYT‐gene mutations (DYT‐THAP1) verified in the extension period negatively impacted DBS effects. None of the non‐responders presented with fixed skeletal deformities as a possible negative outcome parameter.^11^, ^17^ Patients with immediate or delayed non‐response came from different centers, were evenly distributed between segmental and generalized cohorts, and had no documented system or stimulation setting issues.

### 4.3 Adverse Effects

In line with prior studies, new stimulation‐related AEs between 5‐ and 10‐year FU occurred in 42% of all patients. The total number of patients affected with (S)AE and the overall number of (S)AEs decreased from 32 to 16 patients and from 49 to 24 total events when comparing the first 5 years of therapy with the last 5 years. Stimulation parameters remained stable from 5‐year FU to 10‐year FU. Medication levels stayed reduced between the 5‐ and 10‐year treatment phase. The extent to which a possible motivational decrease for patients to report such events, or for their physicians to document them over the long‐term FU period, may represent a confounding factor remains unclear. However, objectively measurable SAEs had also reduced from n = 21 to n = 11 during the last FU period. A consistent observation is that device‐related (S)AEs occurred twice as often during the 6‐months to 5‐years period compared with the 5–10 years FU. Worsening of dystonia and stimulation‐induced AEs were reported relatively evenly between the aforementioned treatment periods. Dysarthria remained the most frequently reported AE at 5‐year FU, mainly in patients with SD.^8^ Back then, the authors had speculated on possible clinical advantages in the detection of stimulation‐associated speech difficulties in SD patients with rather low baseline speech impairments compared with more severely affected GD patients. Attempts to adjust the stimulation parameters to reduce dysarthria were unsuccessful in two patients and led to a deterioration of the underlying dystonia in two others, prompting them to prefer the initial settings. Although dysarthria remained the most frequently reported AE, the total number of patients with dysarthria decreased by the 10‐year FU, in line with the overall reduction of (S)AEs. Bradykinesia and freezing of gait (FOG) have been described as potential stimulation‐associated side effects in pallidal DBS.^37^, ^38^, ^39^, ^40^, ^41^, ^42^, ^43^ At 5‐year FU, one patient experienced FOG that resolved after adjusting stimulation parameters. At 10‐year FU, another patient reported stimulation‐associated bradykinesia that could be reduced by changing DBS settings. Two other patients exhibited generalized bradykinesia during retrospective video analysis. Unfortunately, bradykinesia was not systematically investigated in this cohort. Moreover, bradykinesia is difficult to evaluate, especially in GD. Nevertheless, stimulation‐induced bradykinesia and/or FOG might have contributed to the worsening of disability scores in some patients in the study.

In summary, most patients had a favorable outcome of bilateral pallidal neurostimulation after up to 10 years, resulting in significant improvements in motor symptoms, disability, mood, and reduced medication requirements. To our knowledge, this study represents the longest prospective, multicenter FU of idiopathic GD and SD patients to date. Over the past two decades, significant advances have transformed the landscape of dystonia treatment, offering unprecedented opportunities that were not available when this study commenced. The key takeaways from this study are two‐fold: first, the remarkable safety and enduring clinical benefits of GPI‐DBS in patients who were otherwise refractory to treatment even a decade post‐surgery. Second, the urgent need for comprehensive (para‐)clinical profiling both before surgery and throughout the postoperative treatment phase in order to reduce the high rate of therapeutic failures. Advances in imaging and gene‐based diagnostics, in particular, offer promising avenues for more precise electrode placement, refined DBS programming, and improved identification of non‐responders, enhancing the personalized effects of DBS.

## Author Roles

(1) Research Project: A. Conception and Design, B. Organization, C. Data Acquisition, Analysis, or Interpretation; (2) Statistical Analysis: A. Design, B. Execution, C. Review and Critique; (3) Manuscript Preparation: A. Writing of the First Draft, B. Review and Critique for Important Intellectual Content; C. Final Approval of the Version to be Published; (4) A. Agreement to be Accountable for All Aspects of the Work in Ensuring that Questions Related to its Accuracy or Integrity are Appropriately Investigated and Resolved.

P.K.: 1A, 1C, 3A, 3B, 3C, 4A.

P.M.: 1A, 1C, 3A, 3B, 3C, 4A.

I.M.S.: 1A, 1C, 3A, 3B, 3C, 4A.

F.S.: 1A, 1C, 3A, 3B, 3C, 4A.

G.D.: 1A, 1C, 3A, 3B, 3C, 4A.

R.E.: 1A, 1C, 3C, 4A.

A.S.: 1A, 1C, 3A, 3B, 3C, 4A.

T.W.: 1A, 1C, 3C, 4A.

J.M.: 1A, 1C, 3C, 4A.

J.Ve.: 3A, 3B, 3C, 4A.

W.P.: 1A, 1C, 3A, 3B, 3C, 4A.

G.H.S.: 3A, 3B, 3C, 4A.

J.Ve.: 3A, 3B, 3C, 4A.

N.W.: 1A, 1C, 3A, 3B, 4A.

W.E.: 3A, 3B, 3C, 4A.

T.P.: 3A, 3B, 3C, 4A.

J.U.M.: 3A, 3B, 3C, 4A.

J.Vo.: 1A, 1B, 1C, 3A, 3B, 3C, 4A.

A.A.K.: 1A, 1B, 1C, 3A, 3B, 3C, 4A.

## Financial Disclosures of All Authors (for the Preceding 12 Months)

A.A.K. received speaker's honoraria and consultancies from Medtronic, Boston Scientific, and Stada Pharm outside the submitted work. P.K. received speaker's honoraria from Medtronic and Stadapharm; and is on the Advisory Board of Abbott and Stadapharm, outside the submitted work. J.Vo. reports grants and personal fees from Boston Scientific and Medtronic; personal fees from Newronika, Ceregate, AbbVie, TEVA, and Abbott St. Jude. P.M. received speaker's honoraria from AbbVie, outside the submitted work. I.M.S. received speaker's honoraria from MedTronic, Boston Scientific, and Desitin Pharma outside the submitted work. F.S. received consultancies from Boston Scientific. T.W. has received honoraria from BIAL, AbbVie, Desitin, Pfizer, Britannia, Esteve, and Licher; consultancies from Stadapharm and Phagenesis; and funding from AbbVie and EVER Pharma. J.M. received speaker's honoraria and consultancies from Merz Pharma, outside the submitted work. A.S. received, unrelated to this research, consulting fees from Abbott, Zambon, and AbbVie; and speaker honoraria from BSH Medical Communication, Abbott, Kyowa Kirin, Novartis, AbbVie, Alexion, and GE Healthcare. W.P. reports consultancy and lecture fees in relation to Parkinson's disease drug development programmes from AC Immune, Alterity, AbbVie, BIAL, Biogen, Britannia, Lilly, Lundbeck, Merz, Neuroderm, Roche, Stada, Takeda, UCB, and Zambon, unrelated to this work. G.D. has served as a consultant for Boston Scientific, Cavion, and Functional Neuromodulation; and has received royalties from Thieme publishers. R.E. has no financial disclosures to declare. G.H.S. received speaker's honoraria from Medtronic, Boston Scientific, and Abbott. W.E. received honoraria for lecturing for Sanofi‐Aventis GmbH. N.W. received travel grants and speaker's honoraria from Abbott, MedTronic, Boston Scientific, Stryker, and Takeda Pharma, outside the submitted work. J.U.M. has no financial disclosures to declare. T.P. has no financial disclosures to declare. Ja.Ve. received speaker's honoraria from Abbott, Boston Scientific, and Saluda; and is on the Advisory Board of Abbott, Uniqure, Latus, and VectorY, outside the submitted work.

## Supporting information


**Data S1.** Supplementary material 1: Participant flow diagram from baseline to 10‐year follow‐up.

**Data S2.** Supplementary material 2: Approved study protocol extension and consent notice on extension period (D450/12).

**Data S3.** Supplementary material 3: Scores: Effect of pallidal neurostimulation on psychiatric symptoms and cognition (higher scores indicating more symptoms). Stimulations Parameters: Neurostimulation settings during the 10‐year study period. Medication: Medical treatment during the 10‐year study period. n, number; n.d., not done; n.a., not applicable; STD, standard deviation.

## Data Availability

The data that support the findings of this study are available on request from the corresponding author. The data are not publicly available due to privacy or ethical restrictions.
