# Supplementary material for: Long‐Term Outcomes on Pallidal Neurostimulation for Dystonia: A Controlled, Prospective 10‐Year Follow‐Up
Source: Mov Disord. 2025 Feb 5;40(6):1098–111. doi: 10.1002/mds.30130 (PMC12160999; doi:10.1002/mds.30130)
Supplement: Supplementary file 1 — Data S1. Supplementary material 1: Participant flow diagram from baseline to 10‐year follow‐up. [file MDS-40-1098-s001.pptx]

## Slide 1
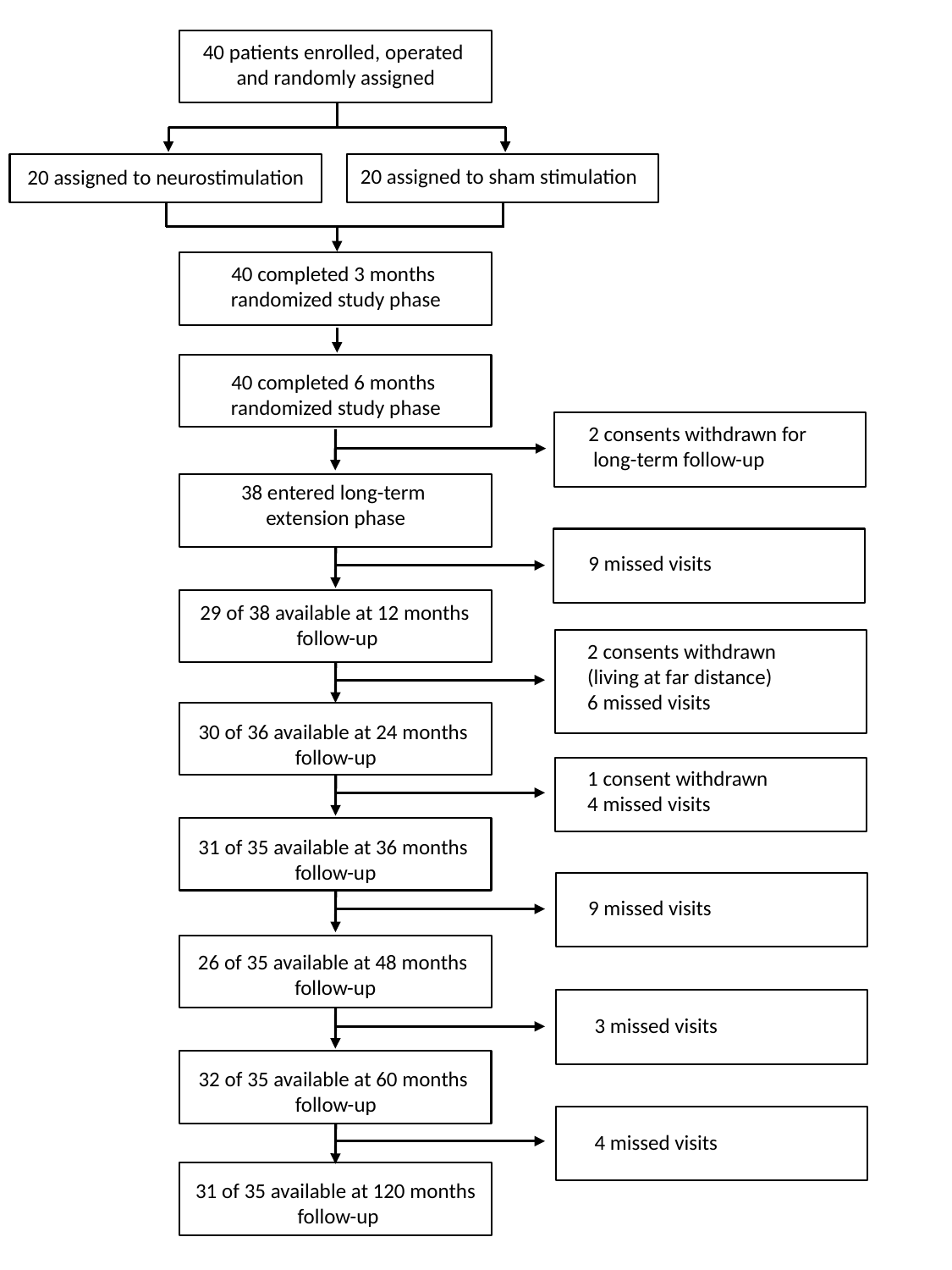

40 patients enrolled, operated
and randomly assigned
20 assigned to sham stimulation
20 assigned to neurostimulation
40 completed 3 months
randomized study phase
40 completed 6 months
randomized study phase
2 consents withdrawn for
 long-term follow-up
38 entered long-term
extension phase
9 missed visits
29 of 38 available at 12 months
follow-up
2 consents withdrawn
(living at far distance)
6 missed visits
30 of 36 available at 24 months
follow-up
1 consent withdrawn
4 missed visits
31 of 35 available at 36 months
follow-up
9 missed visits
26 of 35 available at 48 months
follow-up
3 missed visits
32 of 35 available at 60 months
follow-up
4 missed visits
31 of 35 available at 120 months
follow-up
