# Supplementary material for: Long‐Term Outcomes on Pallidal Neurostimulation for Dystonia: A Controlled, Prospective 10‐Year Follow‐Up
Source: Mov Disord. 2025 Feb 5;40(6):1098–111. doi: 10.1002/mds.30130 (PMC12160999; doi:10.1002/mds.30130)
Supplement: Supplementary file 2 — Data S2. Supplementary material 2: Approved study protocol extension and consent notice on extension period (D450/12). [file MDS-40-1098-s002.pdf]

MEDIZINISCHE FAKULTÄT
DER CHRISTIAN-ALBRECHTS-UNIVERSITÄT ZU KIEL

ETHIK-KOMMISSION

A.D. 1665

Klinik für Neurologie
Universitätsklinikum Schleswig-Holstein
Eing.: 25. Juli 2012
Direktor: Prof. Dr. med. G. Deuschl

Universitäts-Kinderklinik · Schwanenweg 20 · 24105 Kiel

Postadresse:
Arnold-Heller-Straße 3 / Haus 9
D-24105 Kiel

Telefon 04 31 / 597-18 09
Telefax 04 31 / 597-53 33

Herrn
Prof. Dr. med. G. Deuschl
Klinik für Neurologie
im Neurozentrum
Arnold-Heller-Str. 3, Haus 9

24105 Kiel

Datum:

18.7.2012

**AZ: D 450/12**	(bitte stets angeben)
**Studienplan:**	**Langzeitextension (10-Jahres-follow-up) der Studie: Randomisierte, doppelblinde Langzeitstudie zur Wirksamkeit der bilateralen Globus pallidus internus-Stimulation bei idiopathischer generalisierter oder segmentaler Dystonie**
**In Bezug auf:**	
**AZ: A 121/ 02**	
**Studienplan:**	**Randomisierte, doppelblinde Langzeitstudie zur klinischen Wirksamkeit der bilateralen Globus pallidus internus-Stimulation bei idiopathischer generalisierter oder segmentaler Dystonie**
**Antragsteller:**	**PD Dr. J. Volkmann, Klinik für Neurologie, Universitätsklinikum Kiel**
**Datum des Erst-Antrages:**	**05.04.2002**

Ihr Schreiben vom: 10.7.2012 (Eingang 12.7.2012)

Sehr geehrter, lieber Herr Kollege Deuschl,

vielen Dank für Ihren obengenannten Erweiterungsantrag zur Beratung gemäß § 15 Berufsordnung (BO) der Ärztekammer Schleswig-Holstein.

Nach Durchsicht der Unterlagen durch die Geschäftsstelle und durch mich als Vorsitzenden der Ethik-Kommission bestehen gegen die Nachuntersuchung der Patienten im Rahmen der obengenannten Studie keine berufsethischen und berufsrechtlichen Bedenken.

Es wird darauf hingewiesen, dass künftige Änderungen und Erweiterungen des Antrages der Ethik-Kommission anzuzeigen sind und gegebenenfalls eine erneute Beratung erforderlich machen.

Nach Abschluss der Studie erbittet die Kommission einen kurzen Bericht mit einem Hinweis, ob im Laufe der Studie ethische oder juristische Probleme aufgetreten sind.

Mit freundlichen kollegialen Grüßen

Prof. Dr. med. H. M. Mehdorn
Vorsitzender der Ethik-Kommission

Dr. med. Christine Glinicke
Geschäftsführung der Ethik-Kommission

NA

Nachuntersuchung nach 10 Jahren

(Zutreffendes bitte ankreuzen)

Der Nachuntersuchungszeitraum bezieht sich auf das erstmalige Einschalten der chronischen Hochfrequenzstimulation in einem Fenster von 3 Monaten vor bis maximal 18 Monate nach dem jeweiligen Jahrestag.

NA1. Datum der Nachuntersuchung (dd.mm.yy): I__II__I.I__II__I.I__II__I

NA2. Wurde der Patient nachuntersucht? **Ja** **Nein**

(falls nein, füllen Sie bitte das Formblatt „Studienabbruch" aus)

NA3. Gewicht: I__II__II__I kg nicht erhoben

NA4. Blutdruck syst. / diast.: I__II__II__I/I__II__II__I mmHg

NA5. Pulsfrequenz: I__II__II__I

NA6. Stimulation? **Ja** **Nein**

NA7. Sind im Untersuchungszeitraum unerwünschte Ereignisse im Zusammenhang mit der Medikation aufgetreten?

Ja **Nein**

(falls ja, füllen Sie bitte das Formblatt „Nebenwirkungen der medikamentösen Therapie aus")

NA8. Sind im Untersuchungszeitraum Nebenwirkungen in Zusammenhang mit der Stimulationsbehandlung aufgetreten?

Ja **Nein**

(bitte füllen Sie für die operierten Patienten ggf. den Bogen „Nebenwirkungen des Systems" bzw. „Nebenwirkungen der Stimulation" aus).

NA9. Systemkomplikation, die zum Verlust der Stimulationswirkung führt (z.B. Infektion, Kabelbruch, Elektrodendislozierung)?

Ja **Nein**

Falls ja, welche: ____

NA10. Patient verstorben? **Ja** **Nein**

Ursache:____

Nebenwirkungen

(nur einheften falls Nebenwirkungen aufgetreten sind)

NA

NA11 Aktuelle Medikation (Generika)

Anticholinergika	*Beginn (Monat/Jahr)*	*Zahl der Einzeldosen*	*Tagesdosis (mg)*
Trihexyphenidyl			
Biperiden			

Benzodiazepine	*Beginn*	*Einzeldosen*	*Tagesdosis*
Diazepam			
Clonazepam			

Dopaminentspeicherer	*Beginn*	*Einzeldosen*	*Tagesdosis*
Tetrabenazin			

Antispastika	*Beginn*	*Einzeldosen*	*Tagesdosis*
Baclofen			

Neuroleptika	*Beginn*	*Einzeldosen*	*Tagesdosis*
Clozapin			
Pimozid			

L-Dopa + DDCH	*Beginn*	*Einzeldosen*	*Tagesdosis*

NA

NA12 Begleitmedikation - Psychopharmaka

Antidepressiva(Handelsname)	*Wirkstoff*	*Beginn*	*Tagesdosis*

Schlafmittel (Handelsname)	*Wirkstoff*	*Beginn*	*Tagesdosis*

NA13 Begleitmedikation - Sonstige

Schmerzmittel (Handelsname)	*Wirkstoff*	*Beginn*	*Tagesdosis*

Sonstige (Handelsname)	*Wirkstoff*	*Beginn*	*Tagesdosis*

Datum: I__II__I.I__II__I.I__II__I Untersucher:..................

NA

NA14 Botulinumtoxintherapie

(nur auszufüllen bei Injektion innerhalb von 4 Monaten vor Untersuchungsdatum)

1. **Injektionsdatum:** I__I__I.I__I__I. I__I__I__I__I
2. **verwendetes Präparat:** Dysport BOTOX Neurobloc
3. **Injizierte Gesamtdosis:** I__I__I__I__I__I ME
4. **Zielsymptom:** ____
5. **Gesamtanzahl der Injektionsbehandlungen:** I__I__I__I
6. **Abbruch wegen:**

primäres Therapieversagen

sekundäres Therapieversagen:

Krankheitsprogression

Antikörperbildung

Injektionsschema

Muskel	*Menge (ME)*

NA15 Impl. Medikamentenpumpe

Wirkstoff	*Beginn*	*Tagesdosis (mg)*
Lioresal intrathecal		
Morphin intrathecal		

NA

Nebenwirkungen

(nur einheften falls Nebenwirkungen aufgetreten sind)

NA

Studienabbruch

(nur einheften falls Studie abgebrochen wurde)

NA

Stimulationsparameter
(bei Beginn)

1. Wurden die Stimulationsparameter seit der letzten Studienvisite verändert

Nein **Ja**

Wenn **Ja**, Grund der Änderung unzureichende Wirkung

keine
Verstärkung der Dystonie
„Kapselantwort“
Dysästhesien
Phosphene / Gesichtsfeldausfälle
Nausea / Schwindel
____

2. Stimulationsparameter **bei Beginn**

	**Links**	**Rechts**
Normalamplitude	I______I Volt	I______I Volt
Magnetamplitude	I______I Volt	I______I Volt
Frequenz	I______I Hz	I______I Hz
Impulsdauer	I______I µsec	I______I µsec

3. Impedanz gegen IPG case (Nur für **aktive** Elektroden)

EL-0/Case bzw. EL-4	I______I Ohm	I______I Ohm
EL-1/Case bzw. EL-5	I______I Ohm	I______I Ohm
EL-2/Case bzw. EL-6	I______I Ohm	I______I Ohm
EL-3/Case bzw. EL-7	I______I Ohm	I______I Ohm

4. Elektrodenselektion (kreuzen Sie die Polarität für **jede** Elektrode an)

EL-0 bzw. EL-4 neg pos off neg pos
off
EL-1 bzw. EL-5 neg pos off neg pos
off
EL-2 bzw. EL-6 neg pos off neg pos
off
EL-3 bzw. EL-7 neg pos off neg pos
off
Case pos off pos
off

I__II__II__I. I__I I__II__I Langzeitextension
Name. Vorname CRF-Nr.

NA

Videoprotokoll UDRS

	Dauer
**I. AUFNAHME IM SITZEN**	
**1. Augen, obere Gesichtshälfte (Kameraeinstellung auf Augen):**	
a.) Augen geöffnet	10 sec Nahaufnahme,<br>10 sec Gesamtes Gesicht
b.) Augen geschlossen	10 sec Nahaufnahme<br>10 sec Gesamtes Gesicht
c.) Willkürliches Augenblinzeln; 10 Wiederholungen	10 sec
**2. Untere Gesichtshälfte, Kiefer, Zunge, Kehlkopf (Kameraeinstellung auf Gesicht):**	
a.) Nahaufnahme der Gesichtspartie in Ruhestellung	10 sec
b.) Lesen: Lautes Vorlesen des Standardtextes (Thomas Mann „Der Wille zum Glück“ (siehe Anlage))	15 sec
c.) Wiederholung der Silben: Ti, Mi, La, Ka (jeweils 5 Wiederholungen)	15 sec
d.) Den Vokal „iiiii“ 5 sec lang halten	5 sec
e.) Von 1 bis 10 zählen	5 sec
f.) Zunge herausstrecken	5 sec
g.) Mund auf- und zumachen; 5 Wiederholungen	10 sec
**3. Hals (Kameraeinstellung auf Kopf, Hals und Schulterpartie):**	
a.) Sitzen, auf einem Stuhl oder Hocker ohne Rückenlehne, in Frontalansicht	10 sec
b.) Sitzen mit geschlossenen Augen (nach Anweisungen den Kopf bewegen)	10 sec
c.) Ruhige Unterhaltung, die ca. 6 Sätze umfasst	10 sec

NA

d.) Kopf ganz nach rechts und anschließend ganz nach links drehen, Kopf beidseits auf die Schultern legen, nach oben und nach unten schauen	10 sec
e.) Seitenansicht	10 sec

4. Schultern und Oberarme, Unterarme und Hände (Kameraeinstellung auf Oberkörper)

a.) Armhalteversuch in Supinationshaltung	5 sec
b.) Armhalteversuch in Pronationshaltung	5 sec
c.) Armhalteversuch, Beugen und Strecken im Handgelenk beidseitig; 5 Wiederholungen	5 sec
d.) Arme angewinkelt vor der Brust halten	5 sec
e.) Finger-Nase-Versuch; 5 Wiederholungen jeweils rechts und links	5 sec
f.) Fingertapping, erst rechts, dann links: 5 Wiederholungen	5 sec
g.) Becher zum Mund führen, erst mit der rechten, dann mit der linken Hand	5 sec
h.) Schreiben: „Heute ist ein schöner Tag“; 3 Wiederholungen	max 15 sec
i.) Spirale zeichnen ohne die Hand aufzulegen, zuerst mit der rechten, dann mit der linken Hand	max 10 sec
j.) Hochhalten des Blattes mit Spiralzeichnung und der Schreibübung	

5. Becken, Oberschenkel, Unterschenkel, Fuß und Rumpf (Kameraeinstellung auf Unterkörper)

a.) Sitzen in Ruhestellung	10 sec
b.) Fußtapping (abwechselnd Ferse-Zehenspitze): 5 Wiederholungen auf jeder Seite	10 sec

NA

I. AUFNAHME IM STEHEN

1. Hals (Kameraeinstellung auf Kopf, Hals und Schulterpartie)

a.) Gehen (zwei Mal hin und zurück oder 20 sec insgesamt)	20 sec

2. Becken, Oberschenkel, Unterschenkel, Fuß und Rumpf (im Kamerabild Ganzkörpereinstellung)

a.) Stehen: Frontalansicht	10 sec
b.) Stehen: Seitenansicht	5 sec
c.) Stehen: Rückenansicht	5 sec
d.) Gehen: Vom Untersucher ca. 7 m weggehen, umdrehen und zurückkommen; 2 Wiederholungen	20 sec
**Gesamt**	**3 min 05 sec**

NA

Der Wille zum Glück

Der alte Hofmann hatte sein Geld als Plantagenbesitzer Südamerikas verdient. Er hatte dort eine Eingeborene aus gutem Hause geheiratet und war bald darauf mit ihr nach Norddeutschland, seiner Heimat, gezogen. Sie lebten in meiner Vaterstadt, wo auch seine übrige Familie zu Hause war. Paolo wurde hier geboren. Die Eltern habe ich übrigens nicht näher gekannt. Ebenfalls war Paolo das Ebenbild seiner Mutter. Als ich ihn zum ersten Male sah, d.h. als unsere Väter uns zum ersten Male zur Schule brachten, war er ein mageres Bürschchen mit gelblicher Gesichtsfarbe.

(Thomas Mann, Erzählungen)

NA

NA15

BFMDRS motor score

Unterteil Motorik:

Körperregion	Provokations-faktor	x	Schweregrad-faktor	Gewichtungs-faktor	Produkt
Augen	☐		☐	0,5	0-8 ☐
Mund	☐		☐	0,5	0-8 ☐
Sprechen/ Schlucken	☐		☐	1,0	0-16 ☐
Hals	☐		☐	0,5	0-8 ☐
R. Arm	☐		☐	1,0	0-16 ☐
L. Arm	☐		☐	1,0	0-16 ☐
Rumpf	☐		☐	1,0	0-16 ☐
R. Bein	☐		☐	1,0	0-16 ☐
L. Bein	☐		☐	1,0	0-16 ☐
				Summe: (max. = 120)	I__I__I__ I

I. Provokationsfaktoren

A. Allgemein

0 Keine Dystonie in Ruhe oder bei Tätigkeiten

1 Dystonie bei bestimmten Tätigkeiten

2 Dystonie bei vielen Tätigkeiten

3 Bewegungsinduzierte Dystonie an entfernten Körperteilen oder intermittierend in Ruhe

4 Dystonie im Ruhezustand vorhanden

B. Sprechen und Schlucken

1 Gelegentlich bei Sprechen und/oder Schlucken

2 Häufig bei Sprechen oder Schlucken

3 Häufig bei Sprechen und gelegentlich bei Schlucken oder umgekehrt

4 Häufig bei Sprechen und Schlucken

NA

II. Schweregradfaktoren

A. Augen

0 Keine Dystonie vorhanden

1 Schwach. Gelegentliches Blinzeln.

2 Leicht. Häufiges Blinzeln ohne längere Lidkrämpfe

3 Mäßig. Längere Lidkrämpfe, aber Augen sind die meiste Zeit geöffnet

4 Stark. Längere Lidkrämpfe, wobei die Augen mindestens 30% der Zeit geschlossen bleiben

B. Mund

0 Keine Dystonie vorhanden

1 Schwach. Gelegentliches Grimmassieren oder andere Mundbewegungen (z.B. Kieferöffnung oder -schluß; Zungenbewegungen)

2 Leicht. Bewegungen vorhanden in weniger als 50% der Zeit

3 Mäßig ausgeprägte dystone Bewegungen oder Kontraktionen, die meiste Zeit vorhanden

4 Schwer ausgeprägte dystone Bewegungen oder Kontraktionen, die meiste Zeit vorhanden

B. Sprechen und Schlucken

0 Normal

1 Schwach betroffen; Sprache noch gut verständlich oder gelegentliches Verschlucken

2 Sprache etwas schwer zu verstehen oder häufiges Verschlucken

3 Sprache schlecht verständlich oder Unfähigkeit feste Nahrung zu schlucken

4 Vollständige oder fast vollständige Anarthrie oder erhebliche Schluckstörung bei weicher Nahrung oder Flüssigkeiten

NA

**D.**	**Hals**
0	Keine Dystonie vorhanden
1	Schwach. Gelegentliches Verziehen
2	Sichtbarer Torticollis geringer Ausprägung
3	Mäßiges Verziehen
4	Extremes Verziehen

**E.**	**Arm**
0	Keine Dystonie vorhanden
1	Schwache Dystonie, klinisch unbedeutend
2	Leicht. Sichtbare Dystonie, aber nicht behindernd
3	Mäßig. In der Lage zu greifen, Handfunktion teilweise erhalten
4	Schwer. Keine Greiffunktion

**F.**	**Rumpf**
0	Keine Dystonie vorhanden
1	Leichte Rumpfbeugung, klinisch unbedeutend
2	Eindeutige Rumpfbeugung ohne Beeinträchtigung von Stehen oder Gehen
3	Mäßige Rumpfbeugung mit Beeinträchtigung von Stehen oder Gehen
4	Extreme Rumpfbeugung, die Stehen oder Gehen verhindert

**G.**	**Bein**
0	Keine Dystonie vorhanden
1	Schwache Dystonie ohne funktionelle Beeinträchtigung; klinisch unbedeutend
2	Leichte Dystonie. Gehen zügig und ohne Hilfe
3	Mäßige Dystonie. Deutliche Beeinträchtigung beim Gehen oder benötigt Hilfe
4	Schwer: Unfähig zu Stehen oder mit dem betroffenen Bein zu Gehen

NA

NA16 Unterteil Behinderung:

Funktion	Punktzahl
Sprache	0-4 I__I
Schreiben	0-4 I__I
Essen	0-4 I__I
Kauen/Schlucken	0-4 I__I
Hygiene	0-4 I__I
Ankleiden	0-4 I__I
Gehen	0-4 I__I
Summe: (max.=30)	I__II__I

Behinderungsskala

A.	Sprache
0	Normal
1	Schwach betroffen, leicht zu verstehen
2	Etwas schwer verständlich
3	Schwer verständlich
4	Vollständige oder fast vollständige Anarthrie

B.	Handschrift (Tremor oder Dystonie)
0	Normal
1	Leichte Schwierigkeiten; leserlich
2	Fast unleserlich
3	Unleserlich
4	Ist nicht in der Lage zu greifen bzw. einen Stift zu halten

NA

C.	Essen
0	Normal
1	Gebraucht „Tricks“, selbstständig
2	Kann Nahrung mit Löffel oder Gabel zum Mund führen, aber nicht schneiden
3	Isst nur mit Hilfe der Finger
4	Muss gefüttert werden

D.	Kauen / Schlucken
0	Normal
1	Gelegentliches Verschlucken
2	Häufiges Verschlucken; Schwierigkeiten beim Herunterschlucken
3	Ist nicht in der Lage, feste Nahrung zu schlucken
4	Erhebliche Schwierigkeiten beim Schlucken von weicher Nahrung und Flüssigkeiten

E.	Hygiene
0	Normal
1	Unbeholfen; selbstständig
2	Braucht Hilfe bei einigen Tätigkeiten
3	Braucht Hilfe bei den meisten Tätigkeiten
4	Braucht Hilfe bei allen Tätigkeiten

F.	Ankleiden
0	Normal
1	Unbeholfen; selbstständig
2	Braucht Hilfe bei einigen Tätigkeiten
3	Braucht Hilfe bei den meisten Tätigkeiten
4	Hilflos

NA

G.	Gehen
0	Normal
1	gerade abnorm, kaum wahrnehmbar
2	Mäßig abnorm, augenfällig für einen unbefangenen Betrachter
3	Erheblich abnorm
4	Braucht Hilfe beim Gehen
5	Ist auf Rollstuhl angewiesen

Globaler Klinischer Eindruck

NA19 Einschätzung des Patienten:

Schweregrad der Dystonie auf visueller Analogskala:

keine	leicht									schwerst
**0**	**1**	**2**	**3**	**4**	**5**	**6**	**7**	**8**	**9**	**10**

NA20 Einschätzung des Patienten:

Schweregrad der Schmerzen auf visueller Analogskala:

keine	leicht									unerträgli ch
**0**	**1**	**2**	**3**	**4**	**5**	**6**	**7**	**8**	**9**	**10**

NA21 Einschätzung des Untersuchers:

Schweregrad der Dystonie auf visueller Analogskala:

keine	leicht									schwerst
**0**	**1**	**2**	**3**	**4**	**5**	**6**	**7**	**8**	**9**	**10**

I__II__II__I. **I__I** I__II__I Langzeitextension
Name. Vorname CRF-Nr.

NA

NA22 Fragebögen zur Lebensqualität

SF 36

Bitte heften Sie die vom Patienten vollständig ausgefüllten Fragebögen SF 36 hinter dieser Seite ein.

Sollten die Bögen nicht oder unvollständig ausgefüllt sein, geben Sie bitte den Grund dafür an:

	komplett	unvollständig	nicht ausgefüllt	Begründung
SF36				

…………………………

NA

Fragebogen zur Lebensqualität (SF 36)

In diesem Fragebogen geht es um Ihre persönliche Beurteilung Ihres Gesundheitszustandes. Der Bogen ermöglicht es, im Zeitverlauf nachzuvollziehen, wie Sie sich fühlen, und wie Sie im Alltag zurechtkommen.
Bitte beantworten Sie jede der folgenden Fragen, indem Sie bei den Antwortmöglichkeiten die Zahl ankreuzen, die am ehesten für Sie zutrifft.

1. Wie würden Sie Ihren Gesundheitszustand **im allgemeinen** beschreiben?
(Bitte kreuzen Sie nur eine Zahl an.)

Ausgezeichnet	1
Sehr gut	2
Gut	3
Weniger gut	4
Schlecht	5

2. Im Vergleich zum vergangenen Jahr, wie würden Sie Ihren derzeitigen Zustand beschreiben?
(Bitte kreuzen Sie nur eine Zahl an.)

Derzeit viel besser als vor einem Jahr	1
Derzeit etwas besser als vor einem Jahr	2
Etwa so wie vor einem Jahr	3
Derzeit etwas schlechter als vor einem Jahr	4
Derzeit viel schlechter als vor einem Jahr	5

NA

3. Als nächstes sind einige Tätigkeiten beschrieben, die Sie vielleicht an einem normalen Tag ausüben. Sind Sie durch Ihren derzeitigen Gesundheitszustand bei diesen Tätigkeiten eingeschränkt? Wenn ja, wie stark? (Bitte kreuzen Sie nur eine Zahl an.)

**Tätigkeiten**	Ja, stark eingeschränkt	Ja, etwas eingeschränkt	Nein, überhaupt nicht eingeschränkt
**a) anstrengende Tätigkeiten**, z.B. schnell laufen, schwere Gegenstände heben, anstrengenden Sport treiben	1	2	3
**b) mittelschwere Tätigkeiten**, z.B. einen Tisch verschieben, staubsaugen, kegeln, Golf spielen	1	2	3
**c)** Einkaufstaschen heben oder tragen	1	2	3
**d) mehrere** Treppenabsätze steigen	1	2	3
**e) einen** Treppenabsatz steigen	1	2	3
**f)** sich beugen, knien, bücken	1	2	3
**g) mehr als 1 Kilometer** zu Fuß gehen	1	2	3
**h) mehrere Straßenkreuzungen** weit zu Fuß gehen	1	2	3
**i) eine Straßenkreuzung** weit zu Fuß gehen	1	2	3
**j)** sich baden oder ausziehen	1	2	3

4. Hatten Sie **in den vergangenen 4 Wochen** aufgrund Ihrer körperlichen Gesundheit irgendwelche Schwierigkeiten bei der Arbeit oder alltäglichen Tätigkeiten im Beruf, bzw. zu Hause? (Bitte kreuzen Sie nur eine Zahl an.)

**Schwierigkeiten**	**Ja**	**Nein**
**a)** Ich konnte nicht so lange wie üblich tätig sein	1	2
**b)** Ich habe weniger geschafft als ich wollte	1	2
**c)** Ich konnte nur bestimmte Dinge tun	1	2
**d)** Ich hatte Schwierigkeiten bei der Ausführung (z.B. ich mußte mich besonders anstrengen)	1	2

NA

5. Hatten Sie **in den vergangenen 4 Wochen** aufgrund seelischer Probleme irgendwelche Schwierigkeiten bei der Arbeit oder alltäglichen Verrichtungen im Beruf bzw. zu Hause (z.B. weil Sie sich niedergeschlagen oder ängstlich fühlten)? (Bitte kreuzen Sie nur eine Zahl an.)

**Schwierigkeiten**	**Ja**	**Nein**
**a)** Ich konnte nicht so lange wie üblich tätig sein	1	2
**b)** Ich habe weniger geschafft als ich wollte	1	2
**c)** Ich konnte nicht so sorgfältig wie üblich arbeiten	1	2

6. Wie sehr haben Ihre körperliche Gesundheit oder seelischen Probleme **in den vergangenen 4 Wochen** Ihre normalen Kontakte zu Familienangehörigen, Freunden, Nachbarn oder zum Bekanntenkreis beeinträchtigt? (Bitte kreuzen Sie nur eine Zahl an.)

Überhaupt nicht	1
Etwas	2
Mäßig	3
Ziemlich	4
Sehr	5

7. Wie stark waren Ihre Schmerzen in **den vergangenen 4 Wochen**? (Bitte kreuzen Sie nur eine Zahl an.)

Ich hatte keine Schmerzen	1
Sehr leicht	2
Leicht	3
Mäßig	4
Stark	5
Sehr stark	6

NA

8. Inwieweit haben die Schmerzen Sie **in den vergangenen 4 Wochen** bei der Ausführung Ihrer Alltagstätigkeiten zu Hause und im Beruf behindert? (Bitte kreuzen Sie nur eine Zahl an.)

Überhaupt nicht	1
Ein bißchen	2
Mäßig	3
Ziemlich	4
Sehr	5

9. In diesen Fragen geht es darum, wie Sie sich fühlen, und wie es Ihnen **in den vergangenen 4 Wochen** gegangen ist. (Bitte kreuzen Sie nur eine Zahl an.)

Wie oft waren Sie ...

**Befinden**	**Immer**	**Meistens**	**Ziemlich oft**	**Manchma l**	**Selten**	**Nie**
**a)** voller Schwung?	1	2	3	4	5	6
**b)** sehr nervös?	1	2	3	4	5	6
**c)** so niedergeschla-gen, dass Sie nichts aufheitern konnte?	1	2	3	4	5	6
**d)** ruhig und gelassen?	1	2	3	4	5	6
**e)** voller Energie?	1	2	3	4	5	6
**f)** entmutigt und trau-rig?	1	2	3	4	5	6
**g)** erschöpft?	1	2	3	4	5	6
**h)** glücklich?	1	2	3	4	5	6
**i)** müde?	1	2	3	4	5	6

NA

10. Wie häufig haben Ihre körperliche Gesundheit oder seelischen Probleme **in den vergangenen 4 Wochen** Ihre Kontakte zu anderen Menschen beeinträchtigt (Besuche bei Freunden, Verwandten usw.)? (Bitte kreuzen Sie nur eine Zahl an.)

Immer	1
Meistens	2
Manchmal	3
Selten	4
Nie	5

11. Inwieweit treffen die folgenden Aussagen auf Sie zu? (Bitte kreuzen Sie nur eine Zahl an.)

**Aussagen**	**Trifft ganz zu**	**Trifft weit-gehend zu**	**Weiß ich nicht**	**Trifft weit-gehend nicht zu**	**Trifft überhaupt nicht zu**
**a)** Ich scheine etwas leichter als andere krank zu werden	1	2	3	4	5
**b)** Ich bin genauso gesund wie alle anderen, die ich kenne	1	2	3	4	5
**c)** Ich erwarte, daß meine Gesundheit nachläßt	1	2	3	4	5
**d)** Ich erfreue mich ausgezeichneter Gesundheit	1	2	3	4	5

Kompetenznetz Parkinson

NA

NA25 Fragebögen zur Selbstbeurteilung

BAI

Bitte heften Sie die vom Patienten vollständig ausgefüllten Fragebögen BAI und hinter dieser Seite ein.

Sollten die Bögen nicht oder unvollständig ausgefüllt sein, geben Sie bitte den Grund dafür an:

	komplett	unvollständig	nicht ausgefüllt	Begründung
BAI				

…………………………

Summe: (0-63) I__I__I

NA

NA25 Beck Angst Inventar

Auf dieser Seite finden Sie eine Aufstellung von Empfindungen, die vorkommen können, wenn man ängstlich ist. Bitte lesen Sie alles sorgfältig durch. Geben Sie jeweils an, wie sehr Sie durch jede dieser Empfindungen in ***der letzten Woche, einschließlich heute*** belastet waren, indem Sie ein Kreuz in die zutreffende Spalte machen. Bitte vergessen Sie nicht, das heutige Datum einzutragen.

0 Gar nicht
1 Wenig (störte mich nicht sehr)
2 Mittel (Es war sehr unangenehm, aber ich konnte es aushalten
3 Stark (Ich konnte es kaum aushalten)

Datum: I__II__I.I__I.I__I.I__II__I	Gar nicht	Wenig	Mittel	Stark
1. Taubheit oder Kribbeln				
2. Hitzegefühle				
3. Weiche Knie/Beine				
4. Unfähig, mich zu entspannen				
5. Befürchtung des Schlimmsten				
6. Schwindlig oder Benommen				
7. Herzrasen oder –klopfen				
8. Wacklig oder Schwankend				
9. Schrecken				
10. Nervös				
11. Erstickungsgefühle				
12. Zitternde Hände				
13. Zittrig				
14. Angst, die Kontrolle zu verlieren				
15. Atembeschwerden				
16. Angst, zu sterben				
17. Furchtsam				
18. Magen- oder Darmbeschwerden				
19. Schwächegefühl				
20. Glühendes Gesicht				
21. Schwitzen (nicht wegen Hitze)				

NA

NA26 Fragebögen zur Selbstbeurteilung

BDI

Bitte heften Sie die vom Patienten vollständig ausgefüllten Fragebögen BDI hinter dieser Seite ein.

Sollten die Bögen nicht oder unvollständig ausgefüllt sein, geben Sie bitte den Grund dafür an:

	komplett	unvollständig	nicht ausgefüllt	Begründung
BDI				

…………………………

Summe: (0-63) I__I__I

I__II__II__I.   I__I   I__II__I   Langzeitextension
Name.   Vorname   CRF-Nr.

Kompetenznetz Parkinson

NA

Beck Depressions Inventar

Dieser Fragebogen enthält 21 Gruppen von Aussagen. Suchen Sie bitte <u>die</u> Aussage in jeder Gruppe heraus, die am besten beschreibt, wie Sie sich in dieser Woche gefühlt haben und kreuzen Sie dann die dazugehörige Ziffer (0, 1, 2 oder 3) an. Bitte lesen Sie auf jeden Fall zunächst <u>alle</u> Aussagen in jeder Gruppe bevor Sie auswählen.

Datum: I__II__I.I__II__I.I__II__I

**A**	
Ich bin nicht traurig	0
Ich bin traurig	1
Ich bin die ganze Zeit traurig und komme nicht davon los	2
Ich bin so traurig oder unglücklich, daß ich es kaum noch ertrage	3
**B**	
Ich sehe nicht besonders mutlos in die Zukunft	0
Ich sehe mutlos in die Zukunft	1
Ich habe nichts worauf ich mich freuen kann	2
Ich habe das Gefühl, die Zukunft ist hoffnungslos und die Situation kann nicht besser werden	3
**C**	
Ich fühle mich nicht als Versager	0
Ich habe das Gefühl, öfter versagt zu haben als der Durchschnitt	1
Wenn ich auf mein Leben zurückblicke, sehe ich bloß eine Menge Fehlschläge	2
Ich habe das Gefühl, als Mensch ein völliger Versager zu sein	3
**D**	
Ich kann die Dinge genauso genießen wie früher	0
Ich kann die Dinge nicht mehr so genießen wie früher	1
Ich kann aus nichts mehr eine echte Befriedigung ziehen	2
Ich bin mit allem unzufrieden oder gelangweilt	3

NA

**E**	
Ich habe keine Schuldgefühle	0
Ich habe häufig Schuldgefühle	1
Ich habe fast immer Schuldgefühle	2
Ich habe immer Schuldgefühle	3
**F**	
Ich habe nicht das Gefühl, gestraft zu sein	0
Ich habe das Gefühl, vielleicht bestraft zu werden	1
Ich erwarte, bestraft zu werden	2
Ich habe das Gefühl, bestraft zu gehören	3
**G**	
Ich bin nicht von mir enttäuscht	0
Ich bin von mir enttäuscht	1
Ich finde mich fürchterlich	2
Ich hasse mich	3
**H**	
Ich habe nicht das Gefühl, schlechter zu sein als alle anderen	0
Ich kritisiere mich wegen meiner Fehler und Schwächen	1
Ich mache mir die ganze Zeit Vorwürfe wegen meiner Mängel	2
Ich gebe mir für alles die Schuld, was schiefgeht	3
**I**	
Ich denke nicht daran, mir etwas anzutun	0
Ich denke manchmal an Selbstmord, aber ich würde es nicht tun	1
Ich möchte mich am liebsten umbringen	2
Ich würde mich umbringen, wenn ich es könnte	3

NA

**J**	
Ich weine nicht öfter als früher	0
Ich weine jetzt mehr als früher	1
Ich weine die ganze Zeit	2
Früher konnte ich weinen, aber jetzt kann ich es nicht mehr, obwohl ich es möchte	3
**K**	
Ich bin nicht reizbarer als sonst	0
Ich bin jetzt leichter verärgert oder gereizt als früher	1
Ich fühle mich dauernd gereizt	2
Die Dinge, die mich früher geärgert habe, berühren mich nicht mehr	3
**L**	
Ich habe nicht das Interesse an Menschen verloren.	0
Ich interessiere mich jetzt weniger für Menschen als früher.	1
Ich habe mein Interesse an anderen Menschen zum größten Teil verloren.	2
Ich habe mein ganzes Interesse an anderen Menschen verloren.	3
**M**	
Ich bin so entschlussfreudig wie immer.	0
Ich schiebe Entscheidungen jetzt öfter als früher auf.	1
Es fällt mir jetzt schwerer als früher, Entscheidungen zu treffen.	2
Ich kann überhaupt keine Entscheidungen mehr treffen.	3
**N**	
Ich habe nicht das Gefühl, schlechter auszusehen als früher.	0
Ich mache mir Sorgen, daß ich alt oder unattraktiv aussehe.	1
Ich habe das Gefühl, daß in meinem Aussehen Veränderungen eintreten.	2
Ich finde mich häßlich.	3

NA

**O**	
Ich kann so gut arbeiten wie früher.	0
Ich muß mir einen Ruck geben, bevor ich eine Tätigkeit in Angriff nehme.	1
Ich muß mich zu jeder Tätigkeit zwingen.	2
Ich bin unfähig zu arbeiten.	3
**P**	
Ich schlafe so gut wie sonst.	0
Ich schlafe nicht mehr so gut wie früher.	1
Ich wache 1 bis 2 Stunden früher auf als sonst, und es fällt mir schwer, wieder einzuschlafen.	2
Ich wache mehrere Stunden früher auf als sonst und kann nicht mehr einschlafen.	3
**Q**	
Ich ermüde nicht stärker als sonst.	0
Ich ermüde schneller als früher.	1
Fast alles ermüdet mich.	2
Ich bin zu müde, um etwas zu tun.	3
**R**	
Mein Appetit ist nicht schlechter als sonst.	0
Mein Appetit ist nicht mehr so gut wie früher.	1
Mein Appetit hat nachgelassen.	2
Ich habe überhaupt keinen Appetit mehr.	3
**S**	
Ich habe in letzter Zeit abgenommen.	0
Ich habe mehr als 2 Kilo abgenommen.	1
Ich habe mehr als 5 Kilo abgenommen.	2
Ich habe mehr als 8 Kilo abgenommen.	3
Ich esse absichtlich weniger, um abzunehmen Ja [ ] Nein: [ ]	

NA

**T**	
Ich mache mir keine größeren Sorgen um meine Gesundheit als sonst.	0
Ich mache mir Sorgen über körperliche Probleme, wie Schmerzen, Magenbeschwerden oder Verstopfung	1
Ich mache mir so große Sorgen über gesundheitliche Probleme, daß es mir schwerfällt, an etwas anderes zu denken	2
Ich mache mir so große Sorgen über gesundheitliche Probleme, daß ich an nichts anderes mehr denken kann	3
**U**	
Ich habe in letzter Zeit keine Veränderung meines Interesses an Sex bemerkt.	0
Ich interessiere mich weniger für Sex als früher	1
Ich interessiere mich jetzt viel weniger für Sex als früher	2
Ich habe das Interesse an Sex völlig verloren	3

NA

Mattis Demenz Skala

I. Aufmerksamkeit und Konzentration

	Punkte
**A. Zahlenreihen**<br>Vorwärts: 25, 316, 4792<br>Rückwärts: 14, 539, 8593	I__I / (8)
**B. Befolgen von 2 auf einander folgenden Befehlen:**<br>1. Öffnen Sie den Mund und schließen Sie die Augen<br>2. Strecken Sie die Zunge heraus und heben Sie die Hand.<br><br>(1 Punkte für jede richtige Aufgabe)	I__I / (2)

Falls der Patient beide Aufgaben richtig erfüllt,
können C und D übersprungen und maximal bewertet werden.

	Punkte
**C. Befolgen eines verbalen Befehls:**<br>1. Öffnen Sie Ihren Mund<br>2. Strecken Sie die Zunge heraus<br>3. Schließen Sie die Augen<br>4. Heben Sie die rechte Hand<br><br>(1 Punkt für jede richtig ausgeführte Aufgabe)	I__I / (4)
**D. Nachahmen**<br>1. Öffnen Sie Ihren Mund<br>2. Strecken Sie die Zunge heraus<br>3. Schließen Sie die Augen<br>4. Heben Sie die rechte Hand<br><br>(1 Punkt für jede richtig ausgeführte Aufgabe)	I__I / (4)

II/A. verbal

	Punkte
1. Darf ich Sie bitten, alle jene Dinge aufzuzählen, die Sie in einem Supermarkt finden bzw. kaufen können; Nennen Sie so viele verschiedene Dinge wie möglich. Sie haben eine Minute Zeit.<br><br>(Score = Zahl der aufgezählten richtigen Gegenstände in 1 Minute, aber nicht mehr als 20)	I__I / (20)

(Wenn 14 oder mehr der Gegenstände genannt werden, können die Punkte 2, 3 und 4 übersprungen und maximal bewertet werden.)

	Punkte
2. Schauen Sie, wie ich gekleidet bin. Ich würde Sie bitten, alle Kleidungsstücke aufzuzählen, die ich trage. Beispiel geben wie „Mantel, Schuhe" ist erlaubt.<br><br>(Score = Zahl der aufgezählten richtigen Gegenstände in 1 Minute aber nicht mehr als 8)	I__I / (8)
3. Wiederholen Sie bitte „da... sah... nah...".<br>Jetzt sagen sie „da, sah, nah" viermal hintereinander.<br><br>(Score = 1 Punkt für 4 richtige Wiederholungen)	I__I / (1)
4. Wiederholen Sie bitte „da... di... do..."<br>Sagen Sie „da, di, do" viermal hintereinander.<br><br>(Score = 1 Punkt für 4 richtige Wiederholungen)	I__I / (1)

II/B. Motorisch

1. *Zwei gleichzeitig alternierende Bewegungen:*	Punkte
a. Zeigen Sie mit der linken Handfläche nach oben und mit der rechten Handfläche nach unten; dann mehrmals gleichzeitiger Positionswechsel beider Hände. Vormachen erlaubt.<br><br>(Score = 1 Punkt für richtige Änderung der Handstellung bei 5 aufeinander folgenden Wechselbewegungen)	I__I / (1)

Falls der Patient die Aufgabe 1a richtig erfüllt, können 1b und 1c übersprungen werden und maximal bewertet werden

	Punkte
b. Ballen Sie bitte die rechte Hand zur Faust mit der Handfläche nach unten und strecken Sie die Finger der linken Hand (mit der Handfläche nach unten). Ändern Sie die Haltung der Finger (Faustschluss/Fingerstreckung) mehrmals gleichzeitig.<br><br>(Score = 1 Punkt für richtige Änderung der Handstellung bei 5 aufeinander folgenden Wechselbewegungen)	I__I / (1)
c. Bitte klopfen Sie abwechselnd mit den Zeigefingern jeder Hand auf den Tisch. „Klopfen Sie links, dann rechts, dann links, so wie ich jetzt."<br><br>(Score = 1 Punkt für 10 richtige wechselweise Wiederholungen)	I__I / (1)

2. Grafomotorisch (dem Pat. die Vorlage zeigen)	**Punkte**
a. Kopiere 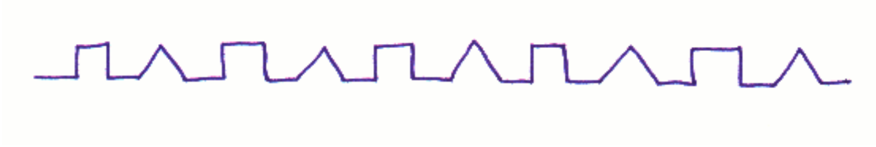	I__I / (1)

Wenn der Patient die Aufgabe 2a richtig erfüllt, können 2b, 2c und 2d übersprungen und maximal bewertet werden.

	**Punkte**
b. Kopiere<br>O	I__I / (1)
c. Kopiere<br>X	I__I /(1)
d. Kopiere<br>OXOXOXOXOXOXOXO	I__I /(1)

III. Konstruktion:

(dem Pat. die Vorlage zeigen)	**Punkte**
A. Kopiere 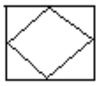	I__I / (1)

Wenn der Patient A richtig löst, können B, C, D, E und F übersprungen und maximal bewertet werden.

	**Punkte**
B. Kopiere 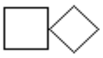	I__I / (1)
C. Kopiere 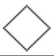	I__I /(1)
D. Kopiere 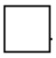	I__I / (1)
E. Kopiere<br>I I I I	I__I /(1)
F. „Schreiben Sie Ihren Namen“	I__I / (1)

IV. Kombinatorik

A. Gemeinsamkeiten

„Auf welche Weise gleichen sich: ... und ...?“

	2 Punkte (abstrakt)	1 Punkt (konkret)	0 Punkte	Punkte
1. Apfel – Banane	beides Früchte	zum Essen,<br>Nahrung,<br>zum Schälen	O	I__I / (2)
2. Mantel - Hemd	beides Kleidungsstücke	halten warm,<br>aus Stoff	O	I__I / (2)
3. Boot – Auto	beides Transportmittel	beide bewegen sich,<br>aus Metall,<br>man kann in/mit beiden fahren	O	I__I / (2)
4. Tisch – Sessel	beides Möbelstücke	4 Beine,<br>stehen in der Küche,<br>aus Holz	O	I__I / (2)

Wenn der Patient 6 oder mehr Punkte bei A erzielt, können B, C, D, E übersprungen und maximal bewertet werden.

B. Instruiertes induziertes Denken

	Punkte
1. Nennen Sie 3 Dinge, die der Mensch **isst**.<br>Antworten: _________, _________, ________<br><br>„ Auf welche Weise gleichen sich ______, ______ und ______?“<br><br>Wenn der Patient nicht oder falsch antwortet, sagen Sie: „_______, _______ und ________ sind alles Dinge, die der Mensch isst.“ (Weiter zu B2)	I__I / (1)
2. Nennen Sie 3 Dinge, die der Mensch **anzieht**.<br><br>Antworten: __________, __________, __________<br>„Auf welche Weise gleichen sich __________, __________ und __________?“<br><br>Wenn der Patient nicht oder falsch antwortet, sagen Sie: „__________, __________ und __________ sind alles Dinge, die Menschen anziehen.“ (Weiter zu B3)	I__I / (1)
3. Nennen Sie 3 Dinge **mit denen man fahren kann**.<br><br>Antworten: __________, __________, __________<br>„ Auf welche Weise gleichen sich __________, __________ und	I__I / (1)

__________?“<br><br>Wenn der Patient nicht oder falsch antwortet, sagen Sie: „__________, __________ und __________ sind alles Dinge, mit denen man fahren kann.“<br><br>(1 Punkt für jede richtige Antwort in Bezug auf die Gemeinsamkeit)	

C. Unterschiede

	Punkte
Ich nenne Ihnen jetzt jeweils 3 Dinge. Sie sagen mir, welches nicht zu den 2 anderen gehört, welches unterschiedlich ist.<br>1. Hund - Katze - Auto<br>2. Junge - Tür - Mann<br>3. Fisch - Auto – Zug<br><br>(1 Punkt für jede richtige Antwort)	I__I / (3)

D. Gemeinsamkeiten (Mehrfachwahl)

	Punkte
1. „Äpfel und Bananen sind das beides Tiere, beides Früchte oder beide grün?“ (Satz kann so oft wie notwendig wiederholt werden)	I__I / (2)*
2. „Mantel und Hemd sind das beides Kleidungsstücke, beide aus Wolle oder beides Früchte?“ (kann wiederholt werden)	I__I / (2)*
3. „Boot und Auto, bewegen sich beide, sind es Transportmittel oder beides Kleidungsstücke?“ (kann wiederholt werden)	I__I / (2)*
4. „Tisch und Sessel, sind das beides Transportmittel, sind beide aus Holz oder beides Möbelstücke?“(kann wiederholt werden)<br><br>* 2 Punkte für jede richtige abstrakte,<br>1 Punkt für jede konkrete Antwort	I__I / (2)*

E. Gleichheit und Ungleichheit

(dem Pat. die Vorlage zeigen)	Punkte
A. „Welche 2 sind gleich, gehören zusammen?“<br>(Zahl der richtigen Antworten)	I__I / (8)
B. „Welches davon ist unterschiedlich, gehört nicht zu den beiden anderen?“<br>(Zahl der richtigen ungleichen, 1 Punkt für jede richtige Antwort)	I__I / (8)

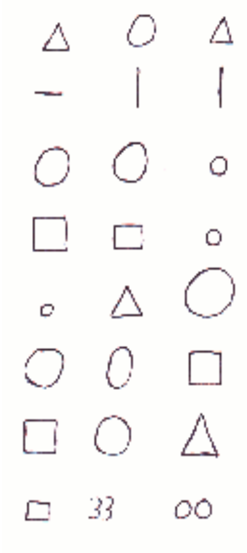

V. Gedächtnis

A1. Verbales Material

Bitten Sie den Patienten, folgenden Satz zu lesen: (dem Pat. die Vorlage zeigen)<br><br>Der Junge hat einen braunen Hund<br><br>Sagen Sie: „Merken Sie sich diesen Satz, weil ich Sie später danach fragen werde.“

B1.

	Punkte
„Ich würde Sie bitten, einen Satz zu bilden, in dem Sie die Wörter - Mann und Auto - verwenden.“<br><br>Antwort: ____<br><br>(1 Punkt für einen vollständigen Satz. Punkt wird der Gruppe Kombinatorik zugezählt - siehe Gesamtbewertungsbogen)<br><br>„Merken Sie sich auch diesen Satz, weil ich später nach ihm fragen werde.“	I__I / (1)

C. Orientierung

	Punkte
Tag, Datum, Monat, Jahr, Präsident, Kanzler, Bürgermeister, Name des Krankenhauses, Stadt<br><br>(1 Punkt für jede richtige Antwort)	I__I / (9)

D1.

(dem Pat. die Vorlage zeigen)	Punkte
„Zählen Sie alle A's"<br>(Ein Punkt für jedes richtig identifizierte A. Punkt wird dem Gebiet Konzentration und Aufmerksamkeit zugezählt - siehe Gesamtbewertungsbogen)	I__I / (6)

D2

(dem Pat. die Vorlage zeigen)	Punkte
(Ein Punkt für jedes richtig identifizierte A. Punkt wird dem Gebiet Konzentration und Aufmerksamkeit zugezählt - siehe Gesamtbewertungsbogen)	I__I / (5)

VI. Satz - Merken - fragen Sie nach den Sätzen

	Punkte
A2. 4 Punkte für den ganzen Satz oder je einen Punkt für Junge, braun und Hund	I__I / (4)
B2. Eigener Satz: 3 Punkte für den kompletten Satz oder 1 Punkt je für Mann und Auto	I__I / (3)

Wiedererkennen von Wörtern
E1

(dem Pat. die Vorlage zeigen)	Punkte
„Ich möchte, dass Sie diese Liste von Wörtern 4mal lesen, so dass Sie sich jedes Wort merken."<br><br>Abend<br>Pflanze<br>offen<br>Maschine<br>Feuer<br><br>(1 Punkt für jedes richtige Lesen aller 5 Wörter. Wird dem Gebiet Konzentration zugezählt)	I__I / (4)

E2

(dem Pat. die Vorlage zeigen)	Punkte
„Ich zeige Ihnen nun jeweils 2 Wörter nebeneinander. Bestimmen Sie bei jedem Wortpaar, welches Wort in der Liste war, die Sie gerade gelesen haben."<br><br>Abend Kopf<br>Meter Pflanze	I__I / (5)

Land offen<br>Maschine Nacht<br>Feuer Milch<br><br>(1 Punkt für jede richtige Antwort)	

F1

(dem Pat. die Vorlage zeigen)	**Punkte**
„Welche dieser Zeichnungen (zeigen Sie auf die untere Reihe) sieht wie diese (zeigen Sie auf die erste Zeichnung aus der oberen Reihe) aus?“<br><br>(Jeder Hinweis erlaubt, der richtiges Erkennen der Übereinstimmung ermöglicht)<br><br>(4 übereinstimmende Zeichnungen, 1 Punkte für jede richtige Übereinstimmung, wird dem Gebiet Aufmerksamkeit und Konzentration zugezählt)	I__I / (4)

F2

(dem Pat. die Vorlage zeigen)	**Punkte**
„Ich zeige Ihnen 2 Zeichnungen gleichzeitig. Bestimmen Sie aus jedem Paar jene Zeichnung mit der Sie gerade gearbeitet haben bzw. die Sie gerade gesehen haben.“<br>(1 Punkt für jede richtige Antwort)	I__I / (4)

**Summe:**	**I__II__II__I / 144**

Kompetenznetz Parkinson

Gesamtbewertungsbogen „Scoring Sheet“

Aufmerksamkeit und Konzentration:	Punkte	Max.	Aufmerksamkeit und Konzentration:	Punkte	Max.
**I** A (Zahlenreihe)	I__I	**8**	**II** A1 (Supermarkt)	I__I	**20**
B (2 Befehle)	I__I	**2**	A2 (Kleidung)	I__I	**8**
C (verbaler Befehl)	I__I	**4**	A3 (da, sah, nah)	I__I	**1**
D (Nachahmen)	I__I	**4**	A4 (da, di, do)	I__I	**1**
			B 1a (Handfläche)	I__I	**1**
			1b (Faust)	I__I	**1**
			1c (Klopfen)	I__I	**1**
**V** D1 (A's zählen)	I__I	**6**	2a (Kopieren)	I__I	**1**
D2 (Aa's zählen)	I__I	**5**	2b (Kopieren O)	I__I	**1**
E1 (Liste lesen)	I__I	**4**	2c (Kopieren X)	I__I	**1**
F1 (Zeichnungen zuordnen)	I__I	**4**	2d (Kopieren OXOX)	I__I	**1**
**Summe**	**I__II__I**	**37**	**Summe**	**I__II__I**	**37**

Konstruktion	Punkte	Max.	Kombinatorik	Punkte	Max.
**III** A (Kopieren)	I__I	**1**	**IV** A (Gemeinsamkeiten)	I__I	**8**
B (Kopieren)	I__I	**1**	B (Denken)	I__I	**3**
C (Kopieren)	I__I	**1**	C (Unterschiede)	I__I	**3**
D (Kopieren)	I__I	**1**	D (Gemeinsamkeiten)	I__I	**8**
E (Kopieren)	I__I	**1**	E (Un-/Gleichheit)	I__I	**16**
F (Name)	I__I	**1**			
			**V** B1 (kompletter Satz)	I__I	**1**
**Summe**	**I__II__I**	**6**	**Summe**	**I__II__I**	**39**

Gedächtnis	Punkte	Max.	Totalscore	Punkte	Max.
**VI** A2 (Junge, braun, Hund)	I__I	**4**	**Aufmerksamkeit und**	**I__II__I**	**37**
B2 (Mann, Auto)	I__I	**3**	**Konzentration**	**I__II__I**	**37**
			**Konstruktion**	**I__I**	**6**
**V** C (Orientierung)	I__I	**9**	**Kombinatorik**	**I__II__I**	**39**
			**Gedächtnis**	**I__II__I**	**25**
**VI** E2 (Wortpaare)	I__I	**5**			
F2 (Zeichnung erkennen)	I__I	**4**			
**Summe**	**I__II__I**	**25**	**Summe**	**I__II__II__I**	**144**

Bemerkungen:

Datum: I__II__I.I__II__I.I__II__I   Untersucher:..................

Brief Psychiatric Rating Scale

Kurzanleitung: Kreuzen Sie das Kästchen an, das den Schweregrad jedes Symptoms beschreibt.

	Nicht vorhanden (1)	Sehr gering (2)	Gering (3)	Mäßig (4)	Mäßig stark (5)	stark (6)	Extrem stark (7)
1. **Köperbezogenheit**<br>Grad der Anteilnahme am augenblicklichen körperlichen Gesundsein. Bewerten Sie, in welchem Ausmaß physische Gesundheit vom Patienten als Problem angesehen wird, gleichgültig, ob ein realer Grund für die Klagen besteht oder nicht.	☐	☐	☐	☐	☐	☐	☐
2. **Angst**<br>Besorgnis, Befürchtungen, Überbesorgnis in Bezug auf Gegenwart und Zukunft. Bewerten Sie nur die verbalen Äußerungen des Patienten über sein subjektives Erleben. Es soll nicht von körperlichen Symptomen oder neurotischen Abwehrmechanismen auf Angst geschlossen werden.	☐	☐	☐	☐	☐	☐	☐
3. **Emotionale Zurückgezogenheit**<br>Mangel an emotionalem Kontakt zum Interviewer und unzureichende Beziehung zur Interviewsituation. Beurteilen Sie lediglich, wie sehr es dem Patienten anscheinend misslingt, emotionalen Kontakt zu anderen Personen in der Interviewsituation herzustellen.	☐	☐	☐	☐	☐	☐	☐
4. **Zerfall der Denkprozesse**<br>Grad, bis zu dem der Denkprozess verworren, inkohärent oder zerfahren ist. Bewerten Sie nur die Integration der verbalen Äußerungen, nicht den subjektiven Eindruck, den der Patient von seinem eigenen Denkvermögen hat.	☐	☐	☐	☐	☐	☐	☐
5. **Schuldgefühle**<br>Überbesorgnis oder Gewissensbisse in Hinsicht auf früheres Verhalten. Bewerten Sie das subjektive Schulderleben aufgrund der verbalen Äußerungen des Patienten und seiner angemessenen affektiven Beteiligung. Es soll nicht von Depression, Angst oder neurotischer Abwehr auf Schuld-gefühle geschlossen werden.	☐	☐	☐	☐	☐	☐	☐
6. **Gespanntheit**<br>Körperlich-motorische Anzeichen für Gespanntheit, "Nervosität" und allgemein erhöhte Aktivität. Bewerten Sie nur die körperlichen Anzeichen von Gespanntheit, nicht das geschilderte subjektive Erleben des Patienten.	☐	☐	☐	☐	☐	☐	☐
7. **Manieriertheit, Affektiertheit, Positur**<br>Auffälligkeiten der Psychomotorik, unübliches motorisches Verhaltensbild, das bestimmte psychisch Kranke aus der Gruppe der "Normalen" heraushebt. Bewerten Sie nur die Abnormität des Bewegungsbildes und der Ausdrucksmotorik, nicht einfach erhöhte motorische Aktivität.	☐	☐	☐	☐	☐	☐	☐

	Nicht vorhanden (1)	Sehr gering (2)	Gering (3)	Mäßig (4)	Mäßig stark (5)	stark (6)	Extrem stark (7)
**8. Größenideen**<br>Überhöhte Selbsteinschätzung, Überzeugung, im Besitz ungewöhnlicher Kräfte und Fähigkeiten zu sein. Bewerten Sie nur die verbalen Äußerungen des Patienten über sich selbst oder im Vergleich zu anderen, nicht jedoch das Verhalten in der Interviewsituation.	□	□	□	□	□	□	□
**9. Depressive Stimmung**<br>Mutlosigkeit, Traurigkeit. Bewerten Sie nur den Grad der Mutlosigkeit. Ziehen Sie keine Rückschlüsse auf Grund von depressiven Begleitsymptomen wie allgemeiner Verlangsamung und körperlichen Beschwerden.	□	□	□	□	□	□	□
**10. Feindseligkeit**<br>Animosität, Geringschätzung, Feindseligkeit, Verachtung gegenüber Personen außerhalb der Interviewsituation. Bewerten Sie nur die verbalen Äußerungen des Patienten über seine Gefühle und Handlungen anderen gegenüber. Es soll nicht von neurotischer Abwehr, Angst oder körperlichen Beschwerden auf Feindseligkeit geschlossen werden. Das Verhalten dem Interviewer ist unter 14 (mangelnde Kooperation) zu bewerten.	□	□	□	□	□	□	□
**11. Misstrauen, paranoide Inhalte**<br>Überzeugung (wahnhaft oder in anderer Weise), dass andere jetzt oder früher böswillige oder diskriminierende Absichten gegenüber dem Patienten haben oder hatten. Bewerten Sie nur solche Verdächtigungen, die aufgrund entsprechender Äußerungen nach wie vor bestehen, gleichgültig ob sie frühere oder derzeitige Situationen betreffen.	□	□	□	□	□	□	□
**12. Halluzinationen**<br>Wahrnehmungen ohne entsprechende normale äußere Reize. Bewerten Sie nur solche Erlebnisse, die laut Patient in der letzten Woche aufgetreten sind und die sich - so wie sie beschrieben werden - deutlich vom Denken und der Vorstellung "Normaler" abheben.	□	□	□	□	□	□	□
**13. Motorische Verlangsamung**<br>Verminderung des Energieniveaus, sichtbar an verlangsamten Bewegungen. Bewerten Sie nur das beobachtete Verhalten des Patienten und nicht den subjektiven Eindruck, den der Patient von seiner Vitalität hat.	□	□	□	□	□	□	□

	Nicht vorhanden (1)	Sehr gering (2)	Gering (3)	Mäßig (4)	Mäßig stark (5)	stark (6)	Extrem stark (7)
**14. Unkooperatives Verhalten** Offensichtlicher Widerstand, Unfreundlichkeit, Vorbehalte und mangelnde Bereitschaft, mit dem Interviewer zusammenzuarbeiten. Bewerten Sie nur die Einstellung des Patienten und seine Reaktionen gegenüber dem Interviewer und auf die Interviewsituation. Beurteilen Sie nicht Äußerungen über ablehnendes oder unkooperatives Verhalten außerhalb der Interviewsituation.	□	□	□	□	□	□	□
**15. Ungewöhnliche Denkinhalte** Ungewöhnliche, seltsame, fremdartige oder bizarre Denkinhalte. Bewerten Sie nur das Ausmaß der Ungewöhnlichkeit, nicht den Grad des Zerfalls der Denkprozesse (formale Denkstörungen sind unter 4 berücksichtigt.).	□	□	□	□	□	□	□
**16. Affektive Abstumpfung, Verflachung** Reduzierte Emotionalität, offensichtlicher Mangel an normalem Fühlen und Engagement.	□	□	□	□	□	□	□
**17. Erregung** Gesteigerte Emotionalität, Agitation, erhöhte Reagibilität.	□	□	□	□	□	□	□
**18. Orientierungsstörungen** Verwirrtheit oder mangelnden Fähigkeit, Personen, Örtlichkeiten oder Zeit zuzuordnen.	□	□	□	□	□	□	□
**Total:**	**I__II__II__I / 126**						

Stimulationsparameter
(bei Entlassung)

1. Datum der Entlassung (dd.mm.yy): I__II__I.I__II__I.I__II__I

2. Stimulationsparameter **bei Entlassung**

	**Links**	**Rechts**
Normalamplitude	I______I Volt	I______I Volt
Magnetamplitude	I______I Volt	I______I Volt
Frequenz	I______I Hz	I______I Hz
Impulsdauer	I______I µsec	I______I µsec

3. Impedanz gegen IPG case (Nur für **aktive** Elektroden)

EL-0/Case bzw. EL-4	I______I Ohm	I______I Ohm
EL-1/Case bzw. EL-5	I______I Ohm	I______I Ohm
EL-2/Case bzw. EL-6	I______I Ohm	I______I Ohm
EL-3/Case bzw. EL-7	I______I Ohm	I______I Ohm

4. Elektrodenselektion (kreuzen Sie die Polarität für **jede** Elektrode an)

EL-0 bzw. EL-4	neg	pos	off	neg	pos	off
EL-1 bzw. EL-5	neg	pos	off	neg	pos	off
EL-2 bzw. EL-6	neg	pos	off	neg	pos	off
EL-3 bzw. EL-7	neg	pos	off	neg	pos	off
Case		pos	off		pos	off
