# Supplementary material for: Long‐Term Outcomes on Pallidal Neurostimulation for Dystonia: A Controlled, Prospective 10‐Year Follow‐Up
Source: Mov Disord. 2025 Feb 5;40(6):1098–111. doi: 10.1002/mds.30130 (PMC12160999; doi:10.1002/mds.30130)
Supplement: Supplementary file 3 — Data S3. Supplementary material 3: Scores: Effect of pallidal neurostimulation on psychiatric symptoms and cognition (higher scores indicating more symptoms). Stimulations Parameters: Neurostimulation settings during the 10‐year study period. Medication: Medical treatment during the 10‐year study period. n, number; n.d., not done; n.a., not applicable; STD, standard deviation. [file MDS-40-1098-s003.pptx]

## Slide 1
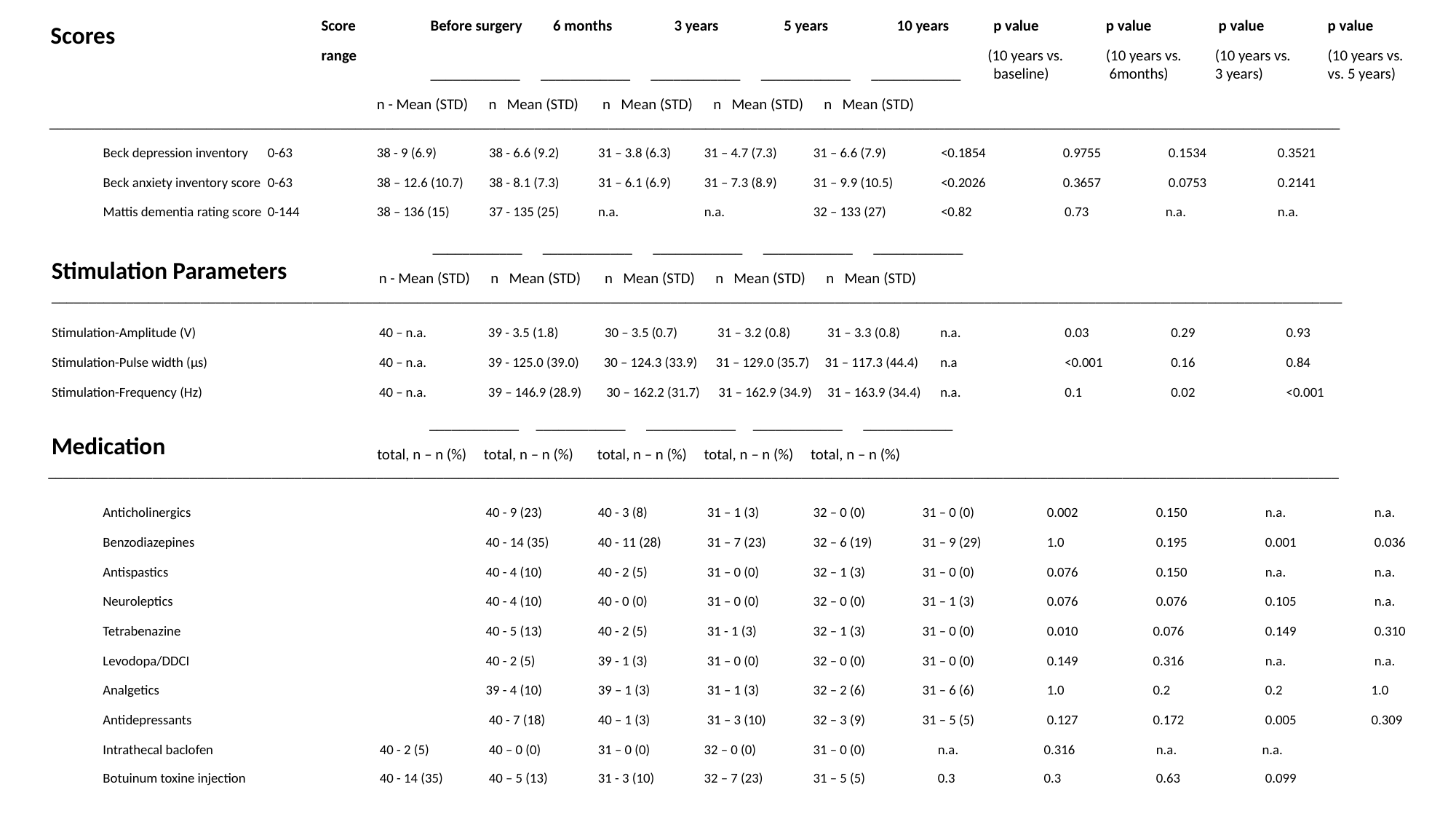

Score 	Before surgery 6 months 3 years 5 years 10 years	 p value	 p value	 p value	 p value
	 	range					 (10 years vs. 	 (10 years vs.	 (10 years vs.	 (10 years vs.		 	____________ ____________ ____________ ____________ ____________ 	 baseline)	 6months)	 3 years)	 vs. 5 years)
		 	n - Mean (STD) n Mean (STD) n Mean (STD) n Mean (STD) n Mean (STD) _____________________________________________________________________________________________________________________________________________________________________________
Beck depression inventory	0-63	38 - 9 (6.9)	 38 - 6.6 (9.2) 	 31 – 3.8 (6.3) 	31 – 4.7 (7.3) 	31 – 6.6 (7.9)	 <0.1854 0.9755	 0.1534	 0.3521
Beck anxiety inventory score 	0-63	38 – 12.6 (10.7)	 38 - 8.1 (7.3) 	 31 – 6.1 (6.9) 	31 – 7.3 (8.9) 	31 – 9.9 (10.5)	 <0.2026 0.3657	 0.0753	 0.2141
Mattis dementia rating score	0-144	38 – 136 (15)	 37 - 135 (25) 	 n.a. 	n.a. 	32 – 133 (27)	 <0.82 0.73	 n.a.	 n.a.
Scores
			____________ ____________ ____________ ____________ ____________
			n - Mean (STD) n Mean (STD) n Mean (STD) n Mean (STD) n Mean (STD) _____________________________________________________________________________________________________________________________________________________________________________
Stimulation Parameters
Stimulation-Amplitude (V) 	40 – n.a. 39 - 3.5 (1.8) 30 – 3.5 (0.7) 31 – 3.2 (0.8) 31 – 3.3 (0.8)	 n.a.	 0.03	 0.29	 0.93
Stimulation-Pulse width (µs)	 	40 – n.a. 39 - 125.0 (39.0) 30 – 124.3 (33.9) 31 – 129.0 (35.7) 31 – 117.3 (44.4)	 n.a	 <0.001	 0.16	 0.84
Stimulation-Frequency (Hz) 	40 – n.a. 39 – 146.9 (28.9) 30 – 162.2 (31.7) 31 – 162.9 (34.9) 31 – 163.9 (34.4)	 n.a.	 0.1	 0.02	 <0.001
			____________ ____________ ____________ ____________ ____________
		 total, n – n (%) total, n – n (%) total, n – n (%) total, n – n (%) total, n – n (%) _____________________________________________________________________________________________________________________________________________________________________________
Medication
Anticholinergics	 		40 - 9 (23)	 40 - 3 (8) 	 31 – 1 (3) 	32 – 0 (0) 	31 – 0 (0)	 0.002	 0.150	 n.a.	 n.a.
Benzodiazepines			40 - 14 (35)	 40 - 11 (28) 	 31 – 7 (23) 	32 – 6 (19) 	31 – 9 (29)	 1.0	 0.195	 0.001	 0.036
Antispastics			40 - 4 (10)	 40 - 2 (5)	 31 – 0 (0) 	32 – 1 (3) 	31 – 0 (0)	 0.076	 0.150	 n.a.	 n.a.
Neuroleptics			40 - 4 (10)	 40 - 0 (0)	 31 – 0 (0) 	32 – 0 (0)	31 – 1 (3)	 0.076	 0.076 	 0.105	 n.a.
Tetrabenazine			40 - 5 (13)	 40 - 2 (5)	 31 - 1 (3) 	32 – 1 (3)	31 – 0 (0)	 0.010	 0.076	 0.149	 0.310
Levodopa/DDCI			40 - 2 (5)	 39 - 1 (3)	 31 – 0 (0) 	32 – 0 (0)	31 – 0 (0)	 0.149	 0.316	 n.a.	 n.a.
Analgetics	 		39 - 4 (10)	 39 – 1 (3)	 31 – 1 (3) 	32 – 2 (6)	31 – 6 (6)	 1.0	 0.2	 0.2	 1.0
Antidepressants			 40 - 7 (18)	 40 – 1 (3)	 31 – 3 (10) 	32 – 3 (9)	31 – 5 (5)	 0.127	 0.172	 0.005	 0.309
Intrathecal baclofen		 40 - 2 (5)	 40 – 0 (0)	 31 – 0 (0) 	32 – 0 (0)	31 – 0 (0)	 n.a.	 0.316	 n.a.	 n.a.
Botuinum toxine injection		 40 - 14 (35)	 40 – 5 (13)	 31 - 3 (10) 	32 – 7 (23)	31 – 5 (5)	 0.3	 0.3	 0.63	 0.099
